# The evaluation of enhanced feedback interventions to reduce unnecessary blood transfusions (AFFINITIE): protocol for two linked cluster randomised factorial controlled trials

**DOI:** 10.1186/s13012-017-0614-8

**Published:** 2017-07-03

**Authors:** Suzanne Hartley, Robbie Foy, Rebecca E. A. Walwyn, Robert Cicero, Amanda J. Farrin, Jill J. Francis, Fabiana Lorencatto, Natalie J. Gould, John Grant-Casey, Jeremy M. Grimshaw, Liz Glidewell, Susan Michie, Stephen Morris, Simon J. Stanworth, for the AFFINITIE programme

**Affiliations:** 1 Clinical Trials Research Unit, University of Leeds, Leeds, UK; 2 Leeds Institute of Health Sciences, University of Leeds, Leeds, UK; 3 Centre for Health Services Research, University of London, London, UK; 4 NHS Blood & Transplant, Oxford, UK; 5 Clinical Epidemiology Program, Ottawa Hospital Research Institute, Ottawa, Canada; 6 Department of Medicine, University of Ottawa, Ottawa, Canada; 7 Centre for Outcomes Research and Effectiveness, University College London, London, UK; 8 Department of Applied Health Research, University College London, London, UK; 9 Transfusion Medicine, NHS Blood and Transplant, Oxford, UK; 10 Department of Haematology, Oxford University Hospitals NHS Foundation Trust, Oxford, UK; 11 Radcliffe Department of Medicine, University of Oxford, and Oxford BRC Haematology Theme, Oxford, UK

**Keywords:** Randomised controlled trial, Cluster randomisation, Split-block design, Implementation, Audit, Blood transfusion

## Abstract

**Background:**

Blood for transfusion is a frequently used clinical intervention, and is also a costly and limited resource with risks. Many transfusions are given to stable and non-bleeding patients despite no clear evidence of benefit from clinical studies. Audit and feedback (A&F) is widely used to improve the quality of healthcare, including appropriate use of blood. However, its effects are often inconsistent, indicating the need for coordinated research including more head-to-head trials comparing different ways of delivering feedback. A programmatic series of research projects, termed the ‘Audit and Feedback INterventions to Increase evidence-based Transfusion practIcE’ (AFFINITIE) programme, aims to test different ways of developing and delivering feedback within an existing national audit structure.

**Methods:**

The evaluation will comprise two linked 2×2 factorial, cross-sectional cluster-randomised controlled trials. Each trial will estimate the effects of two feedback interventions, ‘enhanced content’ and ‘enhanced follow-on support’, designed in earlier stages of the AFFINITIE programme, compared to current practice. The interventions will be embedded within two rounds of the UK National Comparative Audit of Blood Transfusion (NCABT) focusing on patient blood management in surgery and use of blood transfusions in patients with haematological malignancies. The unit of randomisation will be National Health Service (NHS) trust or health board. Clusters providing care relevant to the audit topics will be randomised following each baseline audit (separately for each trial), with stratification for size (volume of blood transfusions) and region (Regional Transfusion Committee). The primary outcome for each topic will be the proportion of patients receiving a transfusion coded as unnecessary. For each audit topic a linked, mixed-method fidelity assessment and cost-effectiveness analysis will be conducted in parallel to the trial.

**Discussion:**

AFFINITIE involves a series of studies to explore how A&F may be refined to change practice including two cluster randomised trials linked to national audits of transfusion practice. The methodology represents a step-wise increment in study design to more fully evaluate the effects of two enhanced feedback interventions on patient- and trust-level clinical, cost, safety and process outcomes.

**Trial registration:**

http://www.isrctn.com/ISRCTN15490813

**Electronic supplementary material:**

The online version of this article (doi:10.1186/s13012-017-0614-8) contains supplementary material, which is available to authorized users.

## Background

Blood transfusion is a common intervention in clinical practice, but transfusions are also a costly and limited resource. The most frequently transfused blood component is red cells, but audits of practice continue to document administration of red cells to groups of stable and non-bleeding patients despite the lack of clear evidence of benefit from clinical trials [1, 2]. Unnecessary transfusion exposes patients to risk, well described by haemovigilance systems such as Serious Hazards of Transfusion (SHOT) in UK, with impacts on mortality and morbidity, through errors in administration and processing, transfusion transmitted infections, acute lung injury and circulatory overload [3, 4].

Active strategies are usually needed to close the gap between recommended and actual clinical practice [5]. Audit and feedback (A&F) is one such widely used approach, defined as a summary of the clinical performance of healthcare providers over a specified period of time [6]. In England, the National Health Service Blood and Transplant (NHSBT) National Comparative Audit (NCA) programme has supported a series of national audits designed to assess whether blood components are used appropriately and safely across clinical specialties [7]. Although participation is voluntary, there are high levels of participation in NCABT audits. Two or three times a year an audit-writing group is assembled, usually comprising an audit lead (typically a consultant haematologist with an interest in transfusion), statistician and clinical staff representatives from the clinical specialty being audited (e.g. orthopaedics). This group agrees upon audit standards against which clinical practice will be compared, the data to be collected and the findings and recommendations to be included in feedback reports. Resulting feedback reports are subsequently made available to the hospital transfusion team (i.e. transfusion practitioner, consultant haematologist, transfusion laboratory manager) via a hospital-specific NCABT audit webpage. Each team is subsequently responsible for disseminating reports within its hospital and, where feedback indicates discrepancies between current practice and audit standards, leading action to improve practice [8].

Despite this rolling programme of national audits, around 20% of transfusions continue to fall outside recommended practice [9, 10], consistent with other international experience [1]. Amongst several possibilities, one likely key explanation for this lack of progress is the variable effectiveness of A&F as an intervention. A Cochrane review of 140 randomised trials found that A&F had modest effects on patient processes of care, leading to a median of 4.3% absolute improvement in compliance with recommended practice (interquartile range 0.5 to 16%). One quarter of A&F interventions had a relatively large, positive effect on quality of care, while another quarter had a negative or null effect. The reasons behind this variation are only partially understood, and further research is needed to improve the consistency and magnitude of the effects of A&F. Furthermore, the relative paucity of head-to-head comparisons of different methods of providing feedback makes it difficult to recommend the use of one feedback strategy over another on empirical grounds [6, 11].

The AFFINITIE programme, ‘Audit and Feedback INterventions to Increase evidence-based Transfusion practice,’ aims to develop and evaluate different ways of delivering feedback embedded in the existing series of audits conducted by the NCABT. The overarching goal is to promote the uptake of evidence-based transfusion guidance and to reduce the unnecessary use of blood components. The AFFINITIE programme follows the UK Medical Research Council (MRC) Framework for the design and evaluation of complex interventions [12] and comprises four work streams with the following objectives:

1. To develop, pilot and refine two feedback interventions, referred to as ‘enhanced content’ and ‘enhanced follow-on support’ [13];
2. To evaluate effectiveness and cost-effectiveness of the two enhanced feedback interventions compared with current standard feedback practice;
3. To investigate the intervention fidelity, including mechanisms of change, for the evaluated interventions [8];
4. To develop general implementation recommendations and tools for relevant A&F programmes in the wider NHS.

This paper describes the second of these work streams, evaluating the effectiveness and cost-effectiveness of enhanced feedback interventions.

## Methods/design

### Study design and setting

**Fig. 1:**
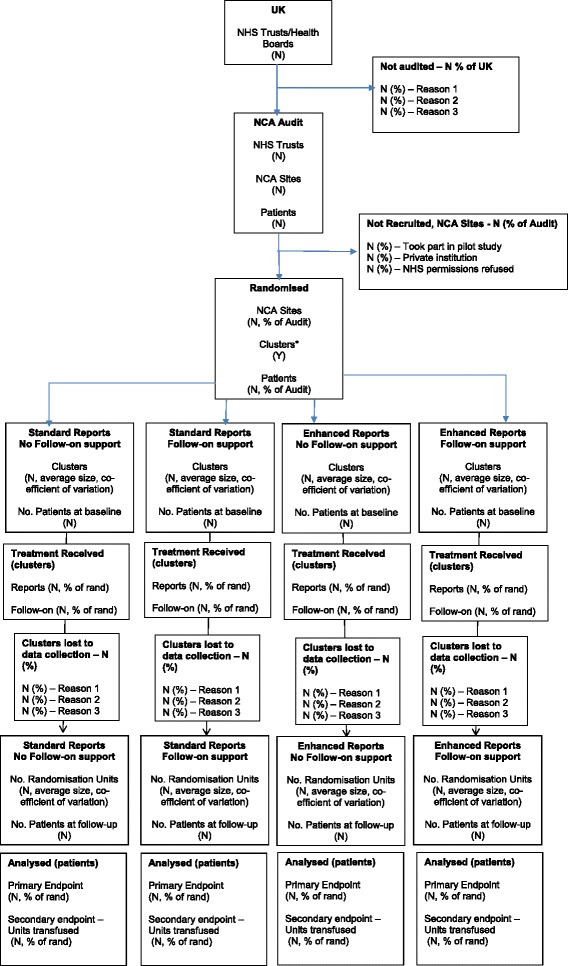
AFFINITIE—CONSORT flow diagram

The evaluation comprises two linked, 2×2 factorial, cross-sectional, cluster-randomised controlled trials (cRCTs) embedded within the NHS NCABT. The two transfusion topics, transfusions in surgical and haematological patients, respectively, were identified and planned by the usual processes conducted by the NCABT. Alongside each audit, we will evaluate two multi-component feedback interventions (enhanced content or enhanced follow-on support) which may be applied singly or in combination; these will be compared with standard feedback, where neither intervention is delivered. Enhanced interventions were developed and piloted in Work Stream 1 of the AFFINITIE Programme based on relevant theory and evidence related to A&F [13]. All feedback, whether enhanced or standard, is directed at clinical teams within hospital trusts and health boards across the UK. The effects of feedback will be assessed using a range of clinical, cost, safety and process end-points. Figure 1 shows the trial CONSORT diagram whilst the full trial protocol is available as Additional file 1.

NHS Trusts and Health Boards can consist of more than one hospital which is covered by a single Hospital Transfusion Team. In addition, several NHS Trusts may share the same Hospital Transfusion Team. Therefore, the unit of randomisation (i.e. cluster) is NHS trust or health board, or group of NHS trusts, to minimise the contamination risk resulting from feedback being directed at a Hospital Transfusion Team that may work across hospitals or NHS Trusts. We adopted, by necessity, a cross-sectional design in which different patients (i.e. cases) are audited at baseline and follow-up (given that it is unlikely that the same patients will receive transfusions during both periods). While there may be some overlap in clinical staff involved in transfusing patients within a cluster over time, a cross-sectional design is assumed here too. Eligible clusters may take part in one or both transfusion audits and associated trials.

### Trial participants

Inclusion criteria are: (i) provision of an NHS service relevant to an audit topic and (ii) acceptance of the invitation by the NCA to participate in the audit. Independent hospitals are not eligible for participation in AFFINITIE, as clinicians involved in transfusion decisions at the NHS trusts and health boards are also likely to practice at independent hospitals, potentially leading to contamination. We will also exclude four NHS trusts that participated in earlier intervention development work. They will still be invited to take part in the national audits and will receive both enhanced feedback interventions.

### Recruitment

#### Clusters

The NCABT will invite NHS trusts and health boards to participate in the audit. The AFFINITIE team will subsequently contact the appropriate transfusion or haematology clinical lead at all sites participating in the audit. This letter will explain that the AFFINITIE program is conducting research to enhance existing quality improvement methods and that involves randomising sites to different types of feedback from the NCABT. We will further explain that the research team will contact the relevant research and development department to seek necessary permissions and that the clinical lead need take no further action. Sites declining participation in the randomised evaluation will be excluded but continue to participate in the NCABT. If the clinical lead has not declined participation within 2 weeks, we will assume that they wish to be included in the study. The AFFINITIE team will seek to obtain permission from the sites that have expressed an interest, or have not declined to participate in the research.

We will document and report reasons for non-participation. Where at least one hospital site within a trust or health board is eligible, the trust or health board will be regarded as eligible. Where multiple hospital sites are eligible within a trust or health board, or where multiple hospitals are known to share a Transfusion Team, the NCABT may treat them as separate but we will regard them as a single cluster for the purposes of randomisation and analysis.

### Randomisation

As part of the 2×2 design, participating trusts and health boards will be randomised to one of four arms: (i) ‘standard content’ and ‘standard follow-on support’; (ii) ‘standard content’ and ‘enhanced follow-on support’; (iii) ‘enhanced content’ and ‘standard follow-on support’; and (iv) ‘enhanced content’ and ‘enhanced follow-on support’. The randomisations will follow baseline data collection in the two audits. The Clinical Trials Research Unit (CTRU) will use an automated system to randomise eligible, consenting trusts and health boards on a 1:1:1:1 basis. A computer-generated minimisation programme, incorporating a random element, will be used to ensure intervention arms are balanced for the following cluster-level characteristics:

- Trust or health board size based on number of audit cases. For each audit, we will review the number of cases submitted to baseline audit per cluster, break these down into thirds (large, medium and small) and later report the final cut-off points used.
- Regional Transfusion Committee (RTC). These cover geographical regions and undertake activities to promote good transfusion practice and to oversee local Hospital Transfusion Committees.

The second transfusion topic (haematology patients) will be used to guide whether the results from the first audit (surgical patients) evaluation can be generalised. We will randomise the four trial arms to clusters separately for each transfusion audit topic, using a “split-block” design [14], rather than keep the allocations the same across topics within trusts/health boards. This design separates the effects of the feedback interventions from time and topic to produce unbiased estimates of effects across trials, increasing the robustness of conclusions drawn from the second trial. The randomised allocation to the first trial will be an additional stratification factor in the randomisation to the second trial. If trusts or health boards merge following randomisation in each trial, they will continue to be regarded as separate and distinct clusters for the intervention, data collection and analysis of that trial. Clusters that merge between trials will be reviewed and a decision made on whether to continue to regard them as distinct clusters in the second trial. Changes will be accounted for in a sensitivity analysis.

Following each randomisation, the CTRU will inform the NCABT, the Chief Investigators and the intervention delivery team of allocations so that appropriate arrangements can be made for intervention delivery as soon as feasible. Other personnel involved in the trial will only be informed of the trial allocation if this is required to undertake their role. We will maintain a log of who is unblinded at specific points in the research process.

### Feedback interventions

#### Standard feedback

Current practice is defined as the standard feedback content and follow-on support delivered by the NCABT following completion of an audit, targeting clinical teams within organisations. Feedback is typically in the form of a written clinical audit report made available via a hospital-specific NCABT audit webpage, a regional PowerPoint presentation and (sometimes) action plan templates. The content of the written report varies, depending on the audit (and will be described in detail). How these clinical teams and organisations respond following receipt of the feedback is regarded as a consequence of the trial interventions—but is presently considered to be variable. We expect them to respond in the context of their clinical governance arrangements. Standard follow-on support involves dealing with data queries from hospitals and is provided by the NCABT Programme Manager. No restrictions will be imposed on current practice or on trusts or health boards undertaking additional development or training in the provision of feedback, with the exception that we will request that the staff who receive the feedback do not share it with colleagues external to their own trust or health board. We will assess and describe the detail of standard feedback content and follow-on within each trial as part of the process evaluation [8].

#### Enhanced content

The enhanced content intervention for each trial has been developed using the current evidence base for A&F [6] and behaviour change theory [15, 16].

The enhanced content concerns the content and format of feedback reports delivered to hospitals and consists of two components. Firstly, the NCABT audit-writing group will receive an *enhancement guidance manual*, which includes guidance on how to apply proposed enhancements for writing feedback reports with evidence- and theory-based content. Secondly, is the resulting *feedback report*, which is uploaded to each hospital’s individual NCABT webpage, where target intervention recipients (i.e. hospital transfusion team) can access and download their feedback reports [8, 13].

The proposed enhancements were identified following a content analysis of previous NCA feedback reports. This examined whether effective components of A&F, identified in the Cochrane Review, and behaviour change techniques (BCTs), consistent with control theory [15] (e.g. goal-setting, feedback, action planning) featured in existing reports [13]. It is intended that the audit-writing group will apply the enhancement guidance manual to produce a template feedback report with enhanced content, which will subsequently be populated with hospital-specific audited data in the feedback report.

Where it is possible we will minimise bias by restricting knowledge of intervention allocation to those who need to know in order to implement the trial.

During intervention development, the members of the enhanced and standard feedback writing groups will be aware which intervention they are developing, however, they will not know which ‘clusters’ are going to receive their intervention until it has been delivered.

Following release of feedback, there is a risk of contamination (e.g. from communication between members of the enhanced and standard feedback report writing groups). However, we will discourage communication pertaining to feedback content between the two writing groups and remind all participants of equipoise (i.e. it is not known whether one feedback method is superior).

#### Enhanced follow-on support

The enhanced follow-on support intervention for each trial concerns the actions taken in hospitals in response to feedback reports. It aims to support relevant hospital transfusion staff response to feedback. It comprises a *web-based toolkit* for use by the hospital transfusion team. The toolkit aims to facilitate three behaviours in response to feedback: dissemination of findings to all relevant clinical staff involved in transfusion decision-making; goal-setting, problem solving and action planning to facilitate practice changes in response to feedback; and continued monitoring of the clinical practices that were audited. The toolkit will be accessible to hospital staff via a web-link uploaded to each hospital’s individual NCABT webpage. As a co-intervention to prompt engagement with the toolkit, hospital transfusion teams will receive an *initial telephone support call* from an intervention facilitator, offering support and advice on how to use the toolkit. A telephone line will subsequently be available for hospitals to contact intervention facilitators for *further support* as needed.

Both types of enhanced feedback can be provided independently as well as in combination, as part of the 2×2 trial design.

### Endpoints

The *primary outcome* for each audit topic is whether a transfusion is categorised as necessary or not (binary measure) and will be measured at the patient level based on the NCABT follow-up audit.

A clinical algorithm for determining if a transfusion is necessary or not will be agreed upon by an independent panel of two clinicians and a statistician, based on clinical relevance and adherence to recommended practice in the baseline audit. The panel will be presented with a description of candidate endpoints, instructed to discuss their clinical relevance and merits, and suggest candidate endpoints to consider further. For these endpoints, we will present the panel with summary information on baseline achievement of the endpoint and the sample size of the final candidate endpoints to ensure that the selected endpoint does not unacceptably reduce the sample size or power below levels allowed for in sample size calculation. The panel members will vote for their preferred outcomes after reviewing the final candidate endpoints. The outcomes with majority support will be selected and documented as the trial primary endpoints, thereby minimising the risk of detection bias.

For the surgical audit, transfusion may occur pre-operatively, intra-operatively or post-operatively. There may also be multiple transfusion episodes after surgery but prior to discharge. As all patients will have had one or more transfusions over the entire operative period (14 days prior to surgery to 7 days following surgery), the primary outcome is whether any of the pre-operative and post-operative transfusions were unnecessary versus all pre-operative and post-operative transfusions being necessary (binary).

For the haematology audit, patients experiencing multiple transfusions of a similar type within the audit period will be audited once only for one of those events. Patient transfused with both red cells and platelets in the audit period will be audited for both. The primary outcome is whether any of these transfusions were unnecessary versus all transfusions being necessary (binary).

For both trials, the Statistical Analysis Plan will specify the statistical programming needed to derive the primary outcome from the patient-level NCABT audit. No clinical judgement will be required at a patient-level to categorise transfusions as necessary or not.

Table 1 outlines the secondary, supportive, intermediate and process level outcomes.

**Table 1:** Secondary, supportive and intermediate outcomes

Surgical audit	
Secondary outcomes	• Total volume of allogeneic red cells transfused (units at trust-level; units at patient-level); • Total number of incidents reported to SHOT (count at trust-level); • Number of definitely, probably or possibly preventable incidents reported to SHOT within clinical specialties targeted by the audit (count at trust-level)
Supportive outcomes	• Pre-operative transfusion (unnecessary/necessary) • Post-operative transfusion (unnecessary/necessary) • Individual NCA audit standard met • Total volume of red cells issued (units at trust-level) • Total volume of red cells wasted (units at trust-level)
Intermediate outcomes (mediators)	Include: • Whether the planned surgery date equals the actual surgery date • Volume of post-operative cell salvage transfused • Hb level • Length of post-operative hospital stay
Trust process level data	• Intervention fidelity • Organizational structures and resources • Tier of data collectors
Haematology audit	
Secondary outcomes	• Total volume of allogeneic red cells transfused (units at trust-level; units at patient-level); • Total volume of platelets transfused (units at patient-level); • Total number of incidents reported to SHOT (count at trust-level); • Number of definitely, probably or possibly preventable incidents reported to SHOT within clinical specialties targeted by the audit (count at trust-level)
Supportive outcomes	• Red cell transfusion alone (unnecessary/necessary) • Platelet transfusion alone (unnecessary/necessary) • Individual NCA audit standard met • Total volume of red cells issued (units at trust-level) • Total volume of red cells wasted (units at trust-level) • Total volume of platelets issued (units at trust-level) • Total volume of platelets wasted (units at trust-level)
Intermediate outcomes (mediators)	Include: • Whether the Hb level was checked after each unit was transfused • Whether the platelet count was measured after each unit was transfused
Trust process level data	• Intervention fidelity • Organizational structures and resources • Tier of data collectors

### Data collection

#### Audit data

Data collected for the NCABT will contribute towards the baseline and follow-up trial data. Existing NCABT procedures for developing a topic-specific audit tool for data collection will be followed. These include convening an Audit Writing Group supported by the NCABT Programme Manager to develop evidence-based audit criteria, ensuring the objectivity of data items collected to minimise observation bias, and incorporating appropriate logic and use of compulsory fields into the online audit tool to maximise the return of complete datasets. The data items collected will depend on the standardised decision algorithm developed for each topic and will include basic patient demographic variables. In line with standard practice, case identification and data collection will be piloted and refined as necessary. Best practice NCABT guidance will be given to trusts and health boards on case identification and data collection, with training recommended and available via the NCABT website. Data collectors will complete the audit tools for all retrievable cases identified by the NCABT.

For each topic, there will be a baseline audit and a follow-up audit approximately 12 months following randomisation. The follow-up audit will include the subset of the items included in the baseline audit required to calculate trial outcomes.

The NCABT will provide the CTRU with four fully anonymised patient-level datasets, covering the baseline and follow-up audits for each of the two transfusion topics. Data will be provided in electronic format via a secure file transfer system. The CTRU will run the end-point algorithms.

Blood Stock Management Scheme (BSMS) collects data in relation to blood stock and wastage management from hospitals in England. The CTRU will request hospital-level datasets which will cover the period 12 months before and 12 months after the feedback is made available to NHS trusts and health boards. The data will be provided in electronic format, via a secure file transfer system, and will include the following variables: month; laboratory; hospital; trust or health board; hospital profile (including electronic/manual cross-matching, cross-match reservation period, cell salvage availability, RTC); blood group; gross issue, net issue, wastage and transfused data for red cells, platelets and adult fresh frozen plasma (FFP).

Serious Hazards of Transfusion (SHOT) is a UK-wide haemovigilance scheme, which collects anonymised data on adverse events and reactions associated with the transfusion of blood and blood components. The CTRU will request fully anonymised patient-level datasets which will cover the period 12 months prior to and 12 months after feedback is made available. The data will again be provided in electronic format, via a secure file transfer system. It will include: incident identifier; patient identifier; speciality; cluster identifier; date of transfusion; blood components transfused and/or implicated component(s); source of component (blood service donor, autologous, directed donation); primary diagnosis for the component transfusion event (adverse event, pathological reaction, transfusion transmitted infection, pulmonary complication of transfusion); type of incident (event, near miss, right blood right patient); date of incident; status (pathological reaction which may not be preventable; probably or possibly preventable by improved practice and monitoring; or adverse event caused by error). For each incident type, data will be collected on investigations, treatments, support and outcomes to facilitate estimates of the costs and health outcomes of transfusion related adverse events for use as inputs into the health economic modelling. We will request this incident data, already stripped of trust and health board identifiers.

The CTRU will use BSMS and SHOT data to derive baseline and outcome variables of interest for the trials (Table 1). Once trust-level datasets have been linked, and prior to performing or reporting any analyses, all identifiers will be removed and trusts and health boards will only be identified by unique consecutive identifiers.

#### Contamination events

There is a potential for contamination of interventions, i.e. site staff receiving standard feedback being exposed to enhanced feedback. Contamination between intervention and control arms may occur at up to six levels:

- Hospital Transfusion Team (e.g. Transfusion Practitioners) communicate with colleagues in other NHS trusts and health boards as part of their role;
- NHS BT Patient Blood Management Practitioners communicate with colleagues in other NHS trusts and health boards;
- Clinical Audit Leads;
- NCABT writing group;
- Clinical staff—junior medical staff training and on rotation between different units and senior medical staff working across different sites;
- NCABT Programme Manager and Statistician;
- The AFFINITIE trial team.

We will assess the respective risks and impacts of contamination at each level. We will take several steps to minimise such risk and we will check for, and monitor, any interactions within the wider AFFINITIE team that may also risk contamination. We will use a combination of brief interviews, observations and diaries to gather data suggesting contamination and to inform interpretation of trial findings.

#### Organisational survey

The NCABT will collect data at timelines corresponding to the baseline and follow-up on structural and resource factors which may influence local adherence to recommended practice (e.g. availability of cell salvage) and to inform the health economic evaluations.

#### Data collectors

Data will be collected on the role of the data collector and will be categorised as follows: tier 1 (Hospital Transfusion Team or Committee); tier 2 (audit department, laboratory); tier 3 (senior clinicians): tier 4 (junior doctors); and tier 5 (nursing staff).

#### Data on intervention delivery

These are described in the process evaluation protocol [8].

#### Resource use and costs

We will collect resource use data to undertake the health economic analyses (Table 2).

**Table 2:** Resource use data to be collected on intervention delivery

Item	Measures of resource use	Additional notes
Audit data collection	Time ﻿of hospital personnel (data collectors) recorded for a sub-sample	Job title and the time taken to extract the audit data from case notes or hospital information systems will be collected on the audit data collection form To minimise burden on sites, the time taken to enter the data onto the NCA system will be estimated by having NCA personnel enter data for a number of ‘mock’ audit cases
Development and delivery of standard content feedback and enhanced content feedback	Time of NCA personnel in designing and populating documents with audit data, including the NCA audit writing groups and associated support	The NCA clinical audit leads and statistician team will record the amount of time taken to perform each of these activities.
Delivery of the enhanced follow-on support	Time of personnel delivering and receiving the enhanced follow-on support, including the web-based toolkit plus telephone support	AFFINITIE team members delivering the enhanced follow-on support will record the duration of telephone support and the use of the online toolkit

### Sample size

For each topic, there are two principal comparisons of interest (enhanced content vs. standard content; and enhanced follow-on support vs. standard follow-on support) relating to the two main effects of the 2×2 factorial design. Assuming 20% unnecessary transfusions at follow-up for each topic [3, 4], an intra-cluster correlation (ICC) of 0.05 and cluster sizes varying from 17 to 68 with a mean of 45, we need 152 clusters to detect a minimally important reduction of 6% (i.e. to 14% unnecessary transfusions) in the presence of, at most, a small antagonistic statistical interaction [17] (i.e. 10% or fewer unnecessary transfusions in the enhanced content and follow-on support) with 80% power using logistic regression models, a random-intercept for cluster, and a 2-sided 2.5% significance level for each comparison within each model. This requires us to recruit from England, Wales, Scotland and Northern Ireland and allows 12/171 (7%) clusters to be ineligible and 95% of those eligible to consent and be randomised. If these assumptions do not all hold, the full trial protocol illustrates the impact on the minimally important clinical difference the trial would be powered to detect (Additional file 1).

### Statistical analysis

#### General considerations

Before any formal analysis, a detailed Statistical Analysis Plan (SAP) will be agreed by the statisticians, the Chief Investigator, other appropriate members of the research team and the Trial Steering Committee.

We expect the proportion of missing data to be non-trivial, making the handling of missing data an important analytical issue. As a sizeable proportion of patients are expected to be missing and the missing data predictable by known variables, we will use multiple imputation under a missing at random (MAR) assumption. Sensitivity analyses will be carried out as appropriate.

As the primary clinical effectiveness analysis for each topic has a single primary outcome but two main treatment effects, two-sided 2.5% significance levels will be used for these contrasts. Where results are subsequently combined when interpreting the treatment effect, we will consider the family-wise error rate and adjustments will be made for multiplicity.

Cluster randomisation imposes recruitment-related clustering. As the impact of clustering is expected to be equal across arms, the principal method for handling this will be to fit a multilevel model that constrains the cluster- and patient-level variances to be equal across arms, that is, a random-intercept model.

No interim analyses are planned. The two audit transfusion topics will be regarded as two trials, but also with a single final analysis when all follow-up data from both topics has been databased, cleaned and locked.

Data distributions will be summarised, cluster and patient-level CONSORT diagrams generated and characteristics of clusters and individuals at baseline and follow-up summarised by arm and by intervention.

The primary analyses will be carried out on an intention-to-treat basis, utilising all available follow-up data from all patients and imputing unavailable follow-up data, comparing allocated interventions. A complier average causal effect (CACE) analysis, comparing treatments received, will be considered if more than 10% of clusters do not implement the intervention as intended.

#### Primary endpoint analysis

For each topic independently, the patient-level binary primary outcome of unnecessary transfusions 12 months following randomisation will be analysed using logistic regression, with a random intercept for trust/health board, adjusting for design factors (that is, trust size, regional transfusion committee), and trust-level proportion of unnecessary transfusions at baseline, with contrasts for: (1) enhanced content vs. standard content; (2) enhanced follow-on support vs. standard follow-on support; and the interaction between (1) and (2), regardless of statistical significance.

#### Secondary endpoint analyses

Patient-level secondary endpoints of volume transfused (both red cells and platelets) will be analysed using a Poisson random-intercept regression model, with the same contrasts and covariates as in the primary endpoint analysis. Trust-level secondary endpoints (volume transfused, number of SHOT-reportable incidents and number of definitely, probably or possibly preventable incidents reported to SHOT within clinical specialities targeted by the audit) will be analysed using cluster-level analyses recognising the outcomes are all counts.

#### Further secondary analyses

Exploratory analyses will be conducted investigating mediators (e.g. fidelity: delivery, receipt and enactment) and moderators (e.g. trust size) of the main effects of the two feedback interventions in the surgical and haematological audits.

A number of exploratory sub-group analyses are planned, which will be specified in detail in the SAP. These include trust and health board and patient level factors such as trust size, Transfusion Practitioner involvement, surgical procedure and haematological diagnosis.

Sensitivity analysis will be undertaken to investigate how contamination events, such as merging hospital trusts, might affect the size and direction of primary outcome measure.

### Process evaluation

In the AFFINITIE programme, the process evaluation will focus on the assessment of fidelity using the fidelity framework proposed by the NIH Behaviour Change Consortium (BCC) [18, 19] to investigate and report the extent to which feedback interventions were designed, trained, delivered, received and enacted as intended. Two linked process evaluations will be conducted alongside the two linked cluster randomised trials [8].

### Economic evaluation

#### Design

Two cost-effectiveness analyses (one for each trial) will be conducted using decision analytic modelling from the perspective of the NHS. We will compare the costs and effects of all four feedback options (standard content feedback, enhanced content only, enhanced follow-on support only and enhanced content and enhanced follow-on support).

#### Methods

Trial-based economic evaluation is not feasible as no unique set of patients is identified at the start of the trial and followed until study completion. Modelling is therefore required to simulate costs and outcomes associated with each option. The models will be developed following a literature review of previously published models of transfusion, and in accordance with good practice guidelines for decision modelling [20].

The models will combine decision tree and Markov approaches and will simulate the main hypothesised costs and effects. These include the costs associated with each intervention, changes in unnecessary transfusions and associated adverse events, plus changes in practice that were introduced to facilitate the change in transfusions.

Resource use data will be collected as specified above. The trials will provide data on the impact of the interventions on transfusions and transfusion-associated adverse events. Data on the volumes of blood components transfused to each audit case will be used to estimate the mean number of product units per unnecessary transfusion. Data from published studies, expert clinical opinion, SHOT and the BSMS will be used to estimate the probabilities, costs, utilities and survival of transfusion-related adverse events. Unit costs will be taken from published sources [21–24].

#### Analysis

We will undertake cost-effectiveness and cost-utility analyses; the former based on primary and secondary outcomes in the trials, the latter based on quality-adjusted life years. A lifetime horizon will be used, with appropriate discounting. We will undertake deterministic and probabilistic sensitivity analysis, the latter assigning appropriate distributions to uncertain model parameters [25].

Cost effectiveness acceptability curves will identify the intervention most likely to be cost-effective for different values of willingness to pay for additional health gain.

### Trial status

The trials are currently in progress. No transfer of endpoint data or cleaning has yet occurred.

## Discussion

The AFFINITIE trials illustrate how to advance scientific knowledge on the effectiveness and cost-effectiveness of A&F through a rigorous evaluation embedded within a national implementation programme. A cumulative meta-analysis of A&F trials indicated that effect sizes stabilised over 10 years ago, suggesting a lack of cumulative learning on how to improve effectiveness [26]. There is now an empirically and theoretically informed research agenda for interventions such as A&F and an acknowledged need to move beyond “business as usual” [27]. This includes undertaking head-to-head trials to compare different approaches to feedback content and delivery, as set out in this protocol.

AFFINITIE is also an example of an ‘implementation laboratory’ that involves close collaboration between a health system delivering an implementation strategy at scale and a research team [11]. This approach offers several mutual advantages. First, by merit of building upon an existing implementation programme and harnessing data already being collected, AFFINITIE will make efficient use of research funding. Second, this close partnership between the NCABT and the research team throughout all stages of the research process will enable knowledge transfer and the subsequent uptake of evidence about implementation into organisational policies and practice [28]. Third, the wide coverage of and high levels of hospital participation in the NCABT will underpin ‘real world’ generalisability. Fourth, it will provide opportunities for parallel mixed-method process evaluations to provide critical insights into mechanisms of change which can inform further research and practice [8].

## Additional file

Additional file 1: Figure S1. CONSORT Flow diagram. (PDF 779 kb)

## Abbreviations

A&F: Audit and feedback
BCT: Behaviour change technique
BSMS: Blood Stock Management Scheme
CONSORT: Consolidated Standards of Reporting Trials
FFP: Fresh frozen plasma
Hb: Haemoglobin
HRQOL: Health-related quality of life
HTT: Hospital Transfusion Team
ICC: Intraclass correlation coefficient
LICTR: Leeds Institute of Clinical Trials Research
NCA: National Comparative Audit
NHS: National Health Service
NHSBT: National Health Service Blood and Transplant
RBC: Red blood cell
RTC: Regional Transfusion Committee
SAP: Statistical Analysis Plan
SHOT: Serious Hazards of Transfusion
TP: Transfusion Practitioner
UK: United Kingdom
