# Supplementary material for: The evaluation of enhanced feedback interventions to reduce unnecessary blood transfusions (AFFINITIE): protocol for two linked cluster randomised factorial controlled trials
Source: Implement Sci. 2017 Jul 3;12:84. doi: 10.1186/s13012-017-0614-8 (PMC5496161; doi:10.1186/s13012-017-0614-8)
Supplement: Additional file 1: Figure S1. — CONSORT Flow diagram. (PDF 779 kb) [file 13012_2017_614_MOESM1_ESM.pdf]

Affinitie

Two linked cluster randomised trials to evaluate feedback interventions embedded within a national audit of transfusion practice

Chief Investigator: Dr Simon Stanworth, Consultant Haematologist, NHS Blood and Transplant, John Radcliffe Hospital, Headley Way, Headington, Oxford, OX3 9BQ

Co-chief investigator: Prof Robbie Foy, Professor of Primary Care, Academic Unit of Primary Care, Leeds Institute of Health Sciences, University of Leeds, 101 Clarendon Road, Leeds, West Yorkshire, LS2 9LJ

Authors: [5]Helen Campbell, [1]Alison Deary, [3]Amanda Farrin, [4]Jill Francis, [2]Liz Glidewell, [4]Natalie Gould, [1]John Grant Casey, [3]Suzanne Hartley, [4]Fabiana Lorencatto, [1]Megan Rowley, [3]Rebecca Walwyn

Collaborating Groups: [1]NHS Blood and Transplant, UK

[2]Leeds Institute of Health Sciences, University of Leeds, UK

[3]Leeds Institute of Clinical Trials Research, University of Leeds, UK

[4]City University London

[5]University of Oxford

University College London

Ottawa Hospital Research Institute, Ottawa, Canada

Protocol version 4.0 06/07/2016
Funder reference: RP-PG-1210-12010
ISRCTN15490813
Research Ethics Committee Reference: 14/EM/1295

The Sponsor accepts no responsibility for the accuracy of content reproduced from this protocol and incorporated into additional documentation developed by collaborating or third party organisations

1 Key Contacts

Chief Investigator
Dr Simon Stanworth
Consultant Haematologist
NHS Blood and Transplant
John Radcliffe Hospital
Oxford OX3 9BQ
Tel: 01865 447976
Email: simon.stanworth@nhsbt.nhs.uk

Co-Chief Investigator
Prof Robbie Foy
Professor of Primary Care,
Academic Unit of Primary Care
Leeds Institute of Health Sciences
University of Leeds
Leeds, LS2 9JL
Tel: 0113 343 4879
Email: r.foy@leeds.ac.uk

Intervention
For queries related to the feedback intervention, contact the Chief Investigator in the first instance.

National Comparative Audit Lead (for queries relating to the Audit)
Mr John Grant-Casey
Programme Manager
National Comparative Audit of Blood Transfusion
NHS Blood and Transplant
John Radcliffe Hospital
Oxford, OX3 9BQ
Tel: 07720 275388
Email: john.grant-casey@nhsbt.nhs.uk

Trial Management (for queries relating to NHS Permissions)
Dr Lauren Moreau
Trial Co-ordinator
Leeds Institute of Clinical Trials Research
Tel: 0113 343 7588
Email: affinitie@leeds.ac.uk

Project Delivery Lead
Ms Suzanne Hartley
Head of Trial Management
Complex Interventions Division
Leeds Institute of Clinical Trials Research
Tel: 0113 343 8041
Email: s.hartley@leeds.ac.uk

Project Scientific Lead
Prof. Amanda Farrin
Professor of Complex Interventions
& Evaluation of Complex Interventions
Leeds Institute of Clinical Trials Research
Tel: 0113 343 8017
Email: a.j.farrin@leeds.ac.uk

Supervising Statistician
Dr. Rebecca Walwyn
Principal Statistician
Complex Interventions Division
Leeds Institute of Clinical Trials Research
Tel: 0113 343 5485
Email: r.e.a.walwyn@leeds.ac.uk

Trial Statistician
Mr Robert Cicero
Medical Statistician
Complex Interventions Division
Leeds Institute of Clinical Trials Research
Tel: 0113 343 8016
Email: r.cicero@leeds.ac.uk

Health Economist

From September 2015
Prof Steve Morris
Chair of Health Economics
Department of Applied Health Research
University College London
Tel: 020 7679 5613
Email: steve.morris@ucl.ac.uk

From project start to September 2015:
Dr Helen Campbell
Senior Researcher
Health Economics Research Centre
Nuffield Department of Population Health
University of Oxford
Tel: 01865 289 274
Email: helen.campbell@dph.ox.ac.uk

Sponsor
NHS Blood and Transplant
Dr Nicholas Watkins
Assistant Director – R&D
c/o National R&D Office
500 North Bristol Park
Northway
Filton
Bristol
BS34 7QH
Tel: +44 (0) 787 263 6715
Email: Research.Office@nhsbt.nhs.uk

Funder
The study is funded as part of a National Institute for Health Research Programme Grant for Applied Research entitled: The development and evaluation of enhanced audit and feedback interventions to increase the uptake of evidence-based transfusion practice (AFFINITIE): Grant reference number RP-PG-1210-12010

Details of AFFINITIE collaborators and grant co-investigators are provided in Appendix A.

2 Contents

1 Key Contacts 2

2 Contents 4

3 Trial Summary 8

3.1 Trial summary flow diagrams 9

3.1.1 Screening / NHS Permission Process 9

3.1.2 Standard NCA Processes / Trial Processes 10

3.2 National Comparative Audit in Blood Transfusion - Clinical audit process flow chart 11

4 Glossary of Terms 12

5 Background 13

5.1 Rationale for study 13

5.2 National Comparative Audits of Blood Transfusion 14

5.2.1 Audit Data Collection Tools 14

5.2.2 Case Identification 15

5.2.3 Audit Data Collection 15

5.2.4 Quality Control 15

5.2.5 Baseline versus Follow-Up Audits 15

6 Aims and objectives of the two trials 17

6.1 Primary Objective of each trial 17

6.2 Secondary Objectives of the trials 17

7 Study design 18

8 Trust (cluster) eligibility 19

8.1 Inclusion criteria: 19

8.2 Exclusion criteria 19

8.3 Clinical audit case selection 20

9 Randomisation of NHS Trusts / Health Boards 21

10 Feedback-related interventions 22
10.1 Usual feedback 22
10.2 Enhanced feedback 22
11 Definition of end of both trials 22
12 Monitoring intervention adherence & fidelity 23
12.1 Process evaluation with sub-sample of hospitals 24
12.1.1 Design 24
12.1.2 Participants and recruitment 24
12.1.3 Procedure 25
12.1.4 Analysis 26
13 Data collection and data transfer 26
13.1 Summary of data collection 27
13.2 Data regarding NHS Trusts / Health Boards 28
13.2.1 Screening and confirmation of eligibility 28
13.2.2 NHS Permissions 28
13.2.3 Blood Stocks Management Scheme 28
13.2.4 Serious Hazard of Transfusion 29
13.2.5 Contamination events 29
13.3 Clinical Audit Data 30
13.3.1 Organisational survey 31
13.4 Data regarding data collectors 32
13.5 Data on intervention delivery 32
13.6 Resource use and costs 33
13.7 Data Transfer 33
14 Withdrawal 34
15 Data monitoring, data management & quality 34
15.1 Monitoring schedule 34
16 Analysis plan and statistical considerations 35

16.1 Endpoints ... 35
16.1.1 Primary endpoint ... 35
16.1.2 Secondary endpoints ... 36
16.1.3 Tertiary endpoints ... 36
16.2 Sample size ... 37
16.3 Statistical analysis ... 38
16.3.1 General considerations ... 38
16.3.2 Frequency of analysis ... 39
16.3.3 Analysis populations ... 39
16.3.4 Primary endpoint analysis ... 39
16.3.5 Secondary endpoint analyses ... 40
16.3.6 Further secondary analyses ... 40
17 Safety ... 41
18 Economic evaluation ... 41
18.1 Design ... 41
18.2 Methods ... 41
18.3 Analysis ... 42
19 Clinical governance issues ... 43
20 Quality assurance & ethical considerations ... 43
20.1 Quality assurance ... 43
20.2 Serious breaches ... 43
20.3 Ethical considerations ... 44
21 Confidentiality ... 45
22 Archiving ... 45
23 Statement of indemnity ... 45
24 Study Organisational Structure ... 45
24.1 Sponsor ... 45
24.2 Chief Investigator ... 46

24.3 Clinical Trials Research Unit (CTRU) ... 46
24.4 Health Economists ... 46
24.5 Staff responsible for Intervention Delivery ... 46
24.6 Audit Leads ... 47
24.7 Data Chasers ... 47
24.8 Data Collectors ... 47
25 Operational Structure ... 47
25.1 Trial Steering Committee (TSC) ... 47
25.2 Trial Management Group (TMG) ... 47
26 Publication policy ... 47
26.1 Principles of Authorship ... 47
26.1.1 Group authorship ... 48
26.1.2 Individual authorship ... 48
26.1.3 Determining authorship and order of authors ... 48
26.2 Authorship for Publications Arising from AFFINITIE ... 49
26.2.1 Operationalising authorship rules ... 49
26.3 Quality assurance ... 49
26.4 Data source ... 50
26.5 Data release ... 50
26.6 NIHR programme grant requirements ... 50
27 References ... 51
Appendix A: Collaborators and grant co-applicants ... 54
Appendix B: Sites involved in intervention development ... 55
Appendix C: Regional Transfusion Committees ... 55

3 Trial Summary

This protocol describes two linked cluster randomised, factorial, cross-sectional trials, each evaluating the effects of two different enhanced feedback interventions, 'enhanced feedback documents' and 'post feedback support' developed and piloted during the first phases of the AFFINITIE research programme. These feedback interventions will be embedded within the current national audit and feedback programme for transfusion. We will compare both interventions against the current standard methods of feedback within the national transfusion audit programme. Each trial will occur in the context of a different audit topic. The two targeted transfusion topics will be (i) patient blood management in surgery and (ii) red blood cell (RBC) and platelet transfusions in haematology patients. These audits will run sequentially. Randomisation will be at the level of hospital NHS trust or Health Board, with stratification for size (volume of blood transfusions) and region (Regional Transfusion Committee). Hospital NHS Trusts and Health Boards will be randomised following each baseline audit for the surgical and haematology patient audit trials respectively. The primary outcome for each topic will be the proportion of patients receiving a transfusion coded as unnecessary. Two cost-effectiveness analyses (one for each audit topic) will be conducted using data generated by the trials as inputs into decision analytic models.

3.1 Trial summary flow diagrams

3.1.1 Screening / NHS Permission Process

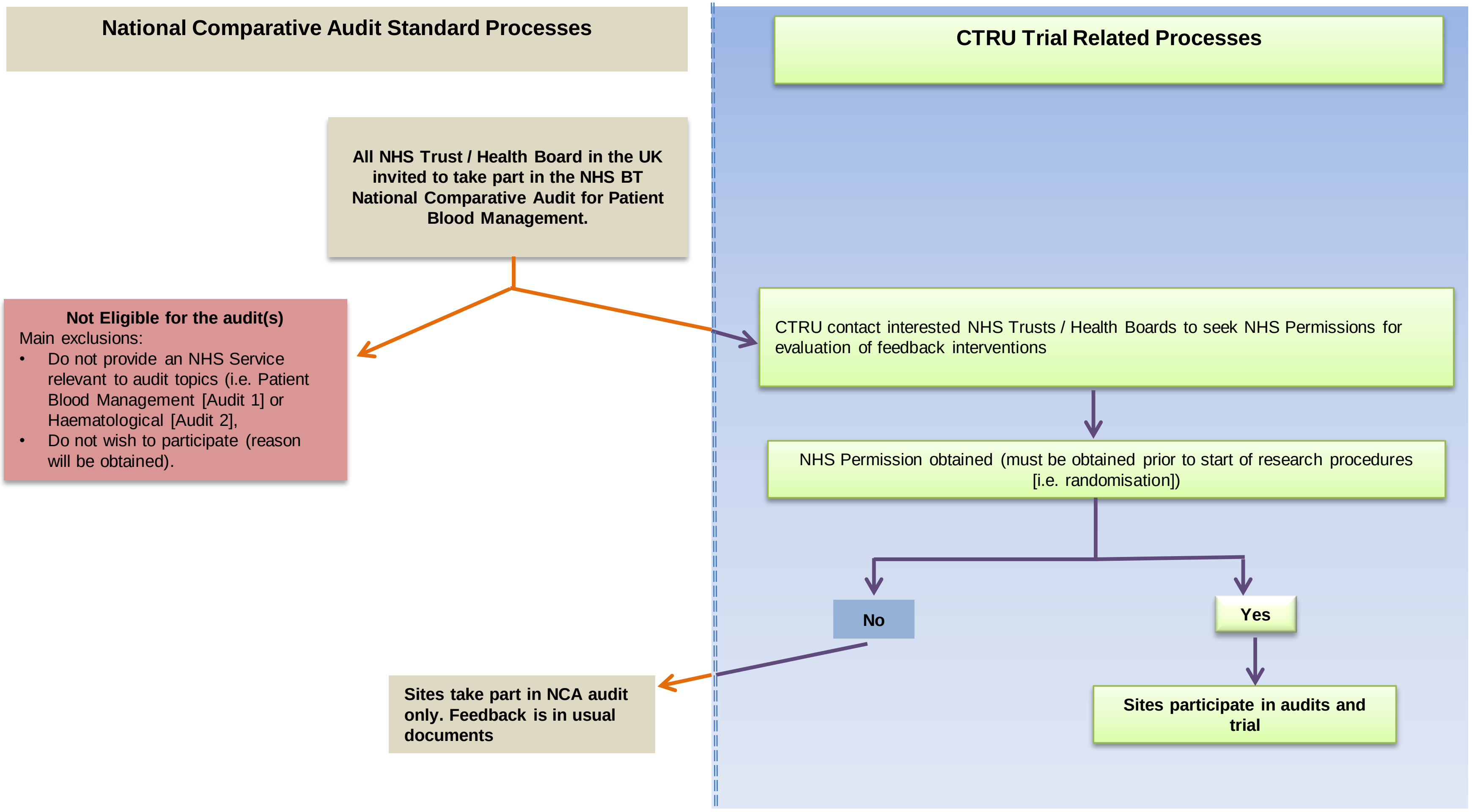

3.1.2 Standard NCA Processes / Trial Processes

* The four NHS Trusts (refer to Appendix B) that participated in the development of the intervention will still be invited to take part in the national audits but will not be randomised and will receive the enhanced feedback documents with post-feedback support. They will therefore not be included in the evaluation of the feedback of post feedback support. This is to prevent contamination whilst still allowing the site to be included in the NCA

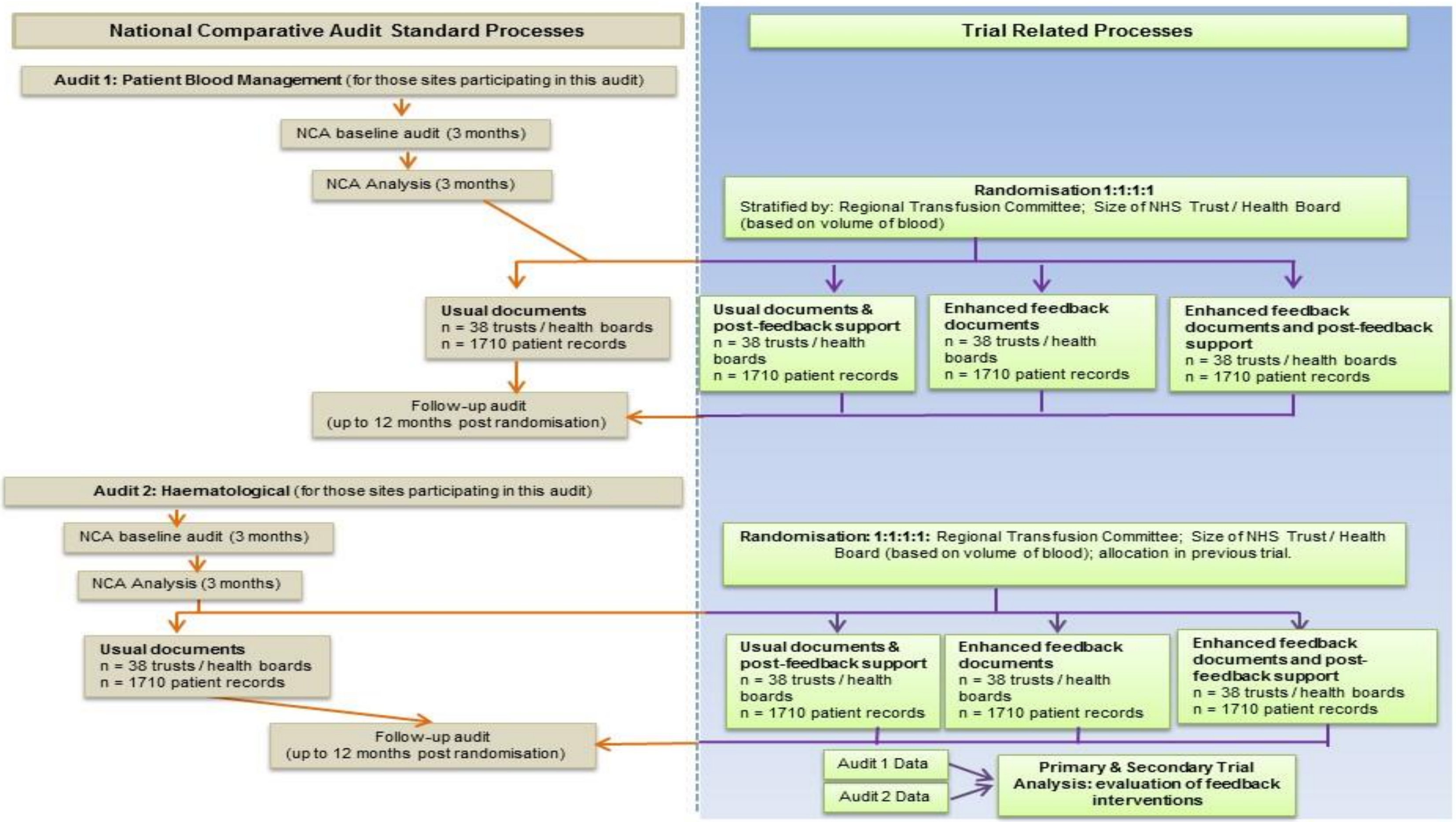

3.2 National Comparative Audit in Blood Transfusion - Clinical audit process flow chart

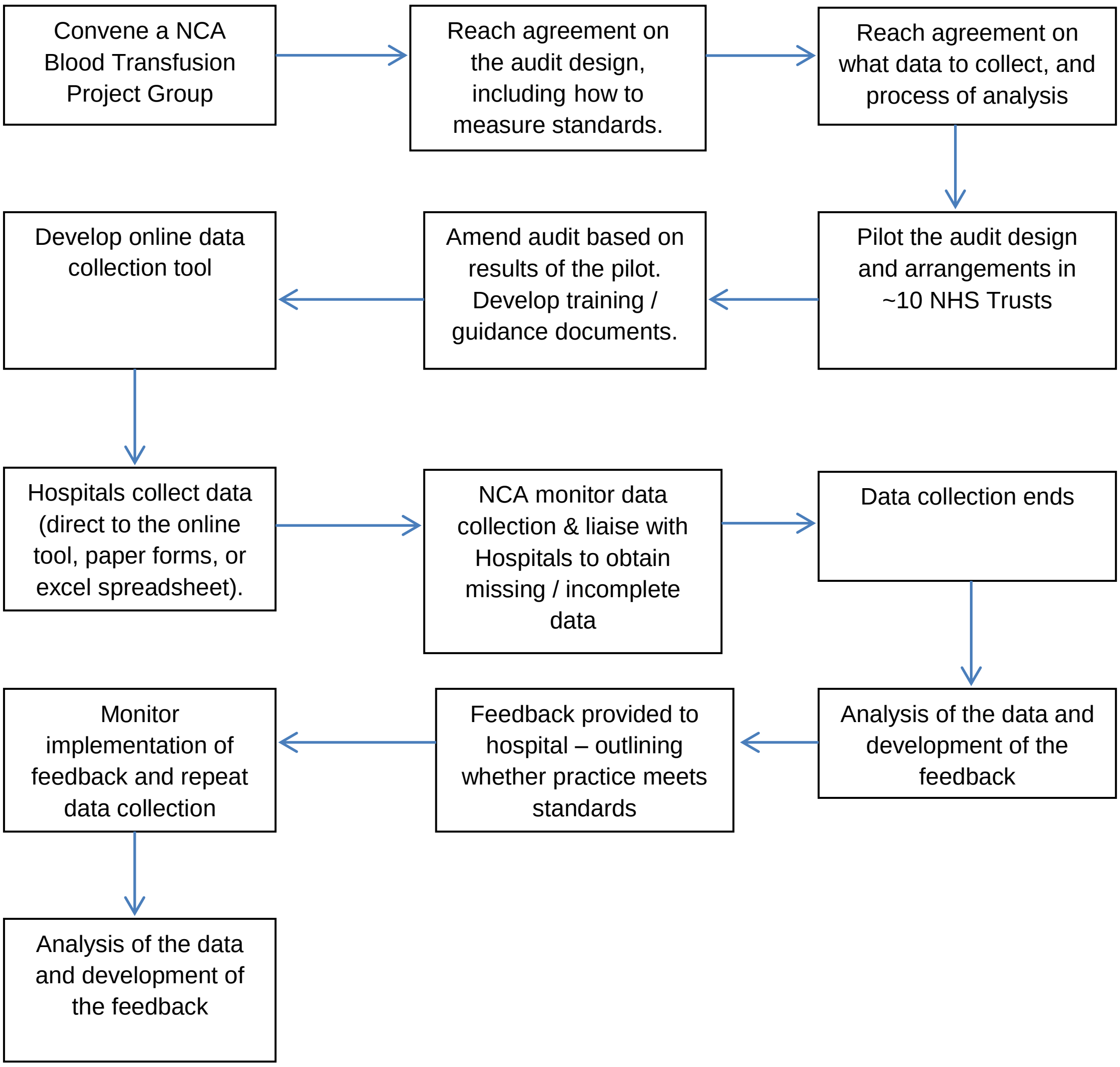

4 Glossary of Terms

AL	Audit Lead
A&F	Audit and Feedback
BCT	Behaviour Change Technique
BSMS	Blood Stock Management Scheme
CI	Chief Investigator
CONSORT	Consolidated Standards of Reporting Trials
CTIMP	Clinical Trial of an Investigative Medicinal Product
CTRU	Clinical Trials Research Unit
DoH	Department of Health
FFP	Fresh Frozen Plasma
GCP	Good Clinical Practice
Hb	Haemoglobin
HRQOL	Health-Related Quality of Life
HTT	Hospital Transfusion Team
ICC	Intraclass Correlation Coefficient
INR	International Normalised Ratio
IT	Information Technology
MRC	Medical Research Council
NCA	National Comparative Audit
NHS	National Health Service
NHSBT	National Health Service Blood and Transplant
NIHR	National Institute for Health Research
NRES	National Research Ethics Service
PT	Prothrombin Time
RBC	Red Blood Cell
REC	Research Ethics Committee
RGF	Research Governance Framework
RTC	Regional Transfusion Committee
SAP	Statistical Analysis Plan
SSA	Site Specific Assessment
SHOT	Serious Hazards of Transfusion
SOP	Standard Operating Procedure
TMG	Trial Management Group
TP	Transfusion Practitioner
TSC	Trial Steering Committee
UK	United Kingdom

5 Background

5.1 Rationale for study

Blood for transfusion is a frequently used clinical intervention in hospital practice. The number of blood components issued by UK transfusion services approaches 3 million/year. Blood for transfusion is also a costly and limited resource. Many transfusions are given to stable and non-bleeding patients where the evidence from clinical studies suggests no clear benefit. In these cases unnecessary or inappropriate transfusion exposes patients to risk. Haemovigilance systems, such as Serious Hazards of Transfusion (SHOT) in the UK, continue to document adverse events impacting upon mortality and morbidity, including errors in the administration, transfusion infections, acute lung injury, circulatory overload, as well as less clearly defined immunomodulatory consequences since blood for transfusion is a biological material [1-2]. For example, recent meta-analyses point to harm from more liberal policies of red cell transfusion, such as hospital acquired infections (Ref JAMA) [3-7].

NHS Blood and Transplant (NHSBT) provides blood components for England and North Wales, and has a programme of support for improving transfusion practice, termed ‘patient blood management’. These activities include national audits of transfusion practice that are undertaken through an on-going collaboration between NHSBT and the Royal College of Physicians, and provide internationally unrivalled data on transfusion practice. National guidelines from within the UK (*British Committee for Standards in Haematology*) and abroad (e.g. American Association of Blood Banks) provide the framework for defining transfusions as ‘unnecessary’ (e.g. by haemoglobin concentrations, platelet count or prothrombin time ratio for transfusions of red cells, platelets and plasma respectively). However, regional and national audits continue to demonstrate at least 20% of red cell use remains outside recommendations and guidelines [8-9]. These data are a consistent finding reported nationally and internationally.

Audit and feedback (defined as any summary of clinical performance of health care over a specified period of time, to provide healthcare professionals with data on performance) is widely used in transfusion (and in many other clinical settings) to improve the quality of NHS care. But there is little rigorous evidence on how to optimise feedback content or delivery, thereby targeting key health professionals or parts of the organisation necessary to support the audit and feedback cycle, reliably maximising effectiveness. Current feedback strategies may not reach either the junior staff who prescribe blood or the senior clinicians who develop local policies. In general, there is a lack of high quality evaluation evidence about methods of enhancing feedback in large audit programmes, and considerable scope exists to improve its impact on patient care.

The AFFINITIE programme focuses on audit and feedback for the following reasons:

- It is widely used and embedded within the NHS, providing an existing vehicle for quality improvement that can be optimised to minimise and/or reduce costs.

- It has variable, small to modest effects across a range of clinical areas, but research is needed on how to enhance effects, including sustainability, compare different ways of providing feedback and understand mechanisms of change.
- A systematic review of quality improvement interventions applied to transfusion [10-11] indicates a dearth of rigorous scientific rationale and evidence to support effectiveness (including by audit and feedback) [12].
- Behavioural theory and research has identified clear pointers for enhancing feedback effectiveness.

This protocol describes two linked and sequential cluster randomised trials embedded within a larger research programme, termed AFFINITIE, funded by NIHR PGfAR (RP-PG-1210-12010). The overarching objective of the AFFINITIE programme is to promote the uptake of evidence-based transfusion guidance, reducing the unnecessary use of blood components. The AFFINITIE programme follows the Medical Research Council (MRC) Framework for the design and evaluation of complex interventions consisting of four work streams:

1. To develop, pilot and refine two feedback-related interventions: loosely referred to as 'enhanced feedback documents' and 'post-feedback support' (Work Stream 1)
2. To evaluate effectiveness and cost-effectiveness of the two feedback interventions compared with current standard feedback practice (Work Stream 2)
3. To investigate the processes of delivery, including mechanisms of change, for the evaluated interventions (Work Stream 3)
4. To develop general implementation recommendations and tools for relevant audit and feedback programmes in the wider NHS (Work Stream 4)

5.2 National Comparative Audits of Blood Transfusion

The two cluster trials are embedded within the existing NCA BT clinical audit programme. The processes followed by the NCA will be maintained, and this section summarises the current processes for undertaking national comparative audits relevant to the trials.

5.2.1 Audit Data Collection Tools

Existing NCA procedures for developing a topic specific audit tool for data collection will be followed (figure 3.2). These include convening a panel of experts to develop evidence-based audit criteria, ensuring the objectivity of data items collected to minimise observation bias, and incorporating appropriate logic and use of compulsory fields into the online audit tool so as to maximise the return of complete datasets. The data items collected will depend on the standardised decision algorithm developed for each topic and will include basic patient demographic variables.

In line with standard practice, the audit collection tool will be piloted in at least 10 NHS Trusts prior to the baseline audit data collection and refined as necessary.

5.2.2 Case Identification

Best practice NCA guidance will be given to Trusts and Health Boards regarding case identification. In addition, this will also allow the trial to minimise the risk of selection bias and allow an assessment of attrition bias. Case Identifiers will generally be Transfusion Practitioners who will work in collaboration with colleagues across the NHS (e.g. informatics) to access hospital lists and databases to identify eligible cases to audit. They will register all consecutive patients meeting eligibility criteria with the NCA. The NCA provide participating Trusts and Health Boards with guidance on how to identify cases. The case identification process will be piloted by the NCA alongside the audit tools.

For the first audit topic, the period of registration will be is open to all NHS Trusts / Health Boards for three months. For the second audit topic, the period of registration is one and a half months.

Full details of the case identification used for each audit will be reported in the Statistical Analysis Plan.

5.2.3 Audit Data Collection

Best practice NCA guidance will be given to Trusts and Health Boards on data collection, with training recommended and available via the NCA website. Each site will select Data Collectors for each topic to: (i) maximise their familiarity with the audit topic and ability to extract maximum accurate data from hospital systems and records; and (ii) minimise their potential role in the feedback interventions. Data Collectors will complete the audit tools for all retrievable cases registered and listed by the NCA.

5.2.4 Quality Control

The quality of data used in the feedback interventions and the research will be maximised by using best practice data management systems and processes, put in place by the NCA. We recognise the risk of intensive ‘chasing’ becoming an unintended intervention in itself and will therefore take steps to ensure this is consistent across all trial arms and undertake it at a level compatible with that of the existing audit. The web-based data entry system will, where possible, include inbuilt warnings flagging illogical dates and data outside expected ranges. NCA Data Chasers will review these warnings and all missing data on a regular basis and will follow them up with identified Data Collectors at Trusts and Health Boards. A trail will be maintained of this process. Trusts and Health Boards will also be given the option to send paper data collection forms or excel spreadsheets to the NCA for data entry.

5.2.5 Baseline versus Follow-Up Audits

For each topic, there will be a baseline audit and a follow-up audit approximately 12 months following randomisation. The follow-up audit will include the subset of the items included in the baseline audit required to calculate outcomes.

5.3 Summary of the two randomised trials

This protocol describes two factorial, cross-sectional, randomised trials, each evaluating the effects of two different enhanced feedback interventions, 'enhanced feedback documents' and 'post feedback support' developed and piloted during the first phases of the AFFINITIE research programme [13]. These feedback interventions will be embedded within the current national audit and feedback programme for transfusion. We will compare both interventions against the current standard methods of feedback within the national transfusion audit programme. Each trial will be applied in the context of a different audit topic. The two targeted transfusion topics will be (i) patient blood management in surgery and (ii) red blood cell (RBC) and platelet transfusions in haematology patients. These audits will run sequentially. Randomisation will be at the level of hospital NHS trust or Health Board, with stratification for size (volume of blood transfusions) and region (Regional Transfusion Committee). Hospital NHS Trusts and Health Boards will be randomised once following each baseline audit for the surgical and haematology patient audit trials, respectively. The primary outcome for each trial will be the proportion of patients receiving a transfusion coded as unnecessary. Two cost-effectiveness analyses (one for each audit topic) will be conducted by using data generated by the trials as inputs into decision analytic models.

6 Aims and objectives of the two trials

6.1 Primary Objective of each trial

The primary objective of each trial is to assess whether (i) ‘enhanced feedback documents’ (enhanced) compared to ‘usual documents’ (usual) and (ii) ‘post-feedback support’ (enhanced) compared to ‘no post-feedback support’ (usual) are more effective in reducing the proportion of patients receiving a transfusion categorised as unnecessary 12 months following randomisation.

6.2 Secondary Objectives of the trials

Secondary objectives are:

- To generate data to serve as inputs for an investigation of the relative cost-effectiveness of the two feedback interventions compared to usual NCA feedback in each audit topic from a NHS perspective

- To investigate whether the two feedback interventions reduce volume of blood products transfused (i) across specialities within NHS trusts and health boards and (ii) for patients treated in specialities targeted by transfusion topics, when compared to usual feedback, 12 months following randomisation

- To investigate whether two feedback interventions reduce the number of errors (defined in the Statistical Analysis Plan) reported to SHOT, when compared to usual feedback, 12 months following randomisation

- To explore whether there are differential predictors (or moderators) of the effects of the two feedback interventions when compared to usual feedback (i.e. subgroup effects)

- To explore the mechanisms by which the two feedback interventions affect outcome (i.e. mediators of the treatment effect)

- To explore whether the effect of the two enhanced feedback interventions when compared to usual feedback differs according to the transfusion topic

7 Study design

The evaluation comprises two multi-centre, 2X2 factorial cross-sectional cluster-randomised, controlled trials, run sequentially, embedded within the NCA, in surgical and haematological patients.

We will evaluate two feedback interventions, directed at clinical teams within hospital trusts / health boards across the UK, developed and piloted as part of the AFFINITIE Programme, comparing them to usual practice, and assessing their effect on patient- and trust-level clinical, cost, safety and process outcomes, to produce robust evidence relating to their effectiveness.

The unit of randomisation is NHS Trust or Health Board to minimise the contamination risks resulting from feedback being directed at a Hospital Transfusion Team who operate at the level of a NHS Trust or Health Board.

We adopted by necessity, at the patient-level, a cross-sectional design in which different patients are audited at baseline and follow-up. While there may be some overlap in clinical staff involved in transfusing patients within a Trust over time, a cross-sectional design is assumed here too. Eligible clusters may take part in one or both of the transfusion topics (i.e. in the surgery or haematological audits) but will be expected to provide baseline and follow-up data (i.e. to take part in the first and last audit for each topic they enrol on).

For each trial, we initially adopted a 2X2 factorial design under the assumption of no statistical interaction in the effect of the two feedback interventions on patient and trust level outcomes. Once the nature of the two feedback interventions became clear, we questioned the likelihood of an additive effect. We gave detailed consideration to the impact of a range of plausible interaction effects on the statistical power available to detect both the main effects of interest and the interaction effect, which is of exploratory interest, and compared to three-arm design alternatives. We proceeded with a 2X2 factorial design because (i) the two comparisons of primary interest are those between each enhanced intervention and its usual comparator, and (ii) it may be argued that we were overly conservative in our initial estimate of the size of the clustering effect. [14] As a consequence, the statistical power to detect the main effects remains fixed at 80% with minimal increases in the minimally important clinical differences that can be detected. The assumptions underlying the sample size calculation will be monitored throughout the project.

As part of the 2X2 design, all trusts and health boards in the UK will be invited to take part. NHS permissions will be sought and consenting clusters will be randomised to one of four interventions: usual documents, usual documents and post-feedback support, enhanced feedback documents, or enhanced feedback documents and post-feedback support following each baseline audit for the surgical and haematology patient audit trials respectively.

Outcomes will be obtained 12 months following each randomisation on an expected number of auditable cases of 45 per cluster but with an anticipated range from 17 to 68. While the target is still to randomise 152 clusters (6840 patients) for each topic, implications of randomising between 130 and 150 clusters have been considered; and the trial would still be

regarded as worthwhile if the target of 152 clusters proved impossible, but at least 130 clusters were included.

The second transfusion topic will be used to determine whether the results can be replicated and generalised. We have decided to randomise the four feedback interventions to clusters separately for each topic using a “split-block” type of design, [24] rather than keep the allocations the same across topics within trusts, to ensure that our estimates of their effects are unbiased across trials, relying on the design to separate the effects of the feedback interventions from time and topic, increasing the robustness of the conclusions drawn about the second trial. Outcome results for the first trial will not be made available outside of the statistical team until results of the second trial are also available.

8 Trust (cluster) eligibility

All NHS Trusts and Health Boards in the UK will be considered for inclusion and invited by the NCA to participate in the audit. The AFFINITIE trial team will subsequently contact the appropriate transfusion or haematology clinical lead at all NHS Trust and Health Boards participating in the audit. This letter will explain that we are, through AFFINITIE, conducting research to enhance existing quality improvement methods and that this will involve randomising organisations to receive different types of feedback from the NCA. We will further explain that the AFFINITIE trial team will contact the relevant R&D department to seek necessary permissions and that the clinical lead need take no further action. However, if s/he has any objections to her/his organisation taking part in the randomised evaluation, or has any questions, s/he can contact us by phone or email. Any organisations which subsequently do not wish to participate in the randomised evaluation will be excluded. If the clinical lead has not indicated that they do not want to take part in the evaluation within 2 weeks, it will be assumed that they wish to be included in the evaluation of the feedback.

Eligibility in the evaluation of the feedback will be based on the following:

8.1 Inclusion criteria:

- Provide an NHS service relevant to audit topics
- Accept the invitation by the NCA to participate in the audit.
- Receive NHS permissions (or equivalent) which will provide evidence of consent at an NHS Trusts / Health Boards ‘cluster’ level to be allocated at random to receive a ***feedback*** intervention under evaluation. The NHS permission provided will cover inclusion in evaluation of feedback for both audit topics.

8.2 Exclusion criteria

- Independent Hospitals will not be eligible for participation as clinicians involved in transfusion decisions at the NHS Trusts / Health Boards are also likely to practice at the independent Hospitals leading to potential contamination.
- The four NHS Trusts (refer to Appendix B) that participated in the development of the intervention will still be invited to take part in the national audits but will not be randomised and will receive the enhanced feedback documents with post-feedback support. They will therefore not be included in the evaluation of the feedback of post

feedback support. This is to prevent contamination whilst still allowing the site to be included in the NCA.

Reasons for non-participation will be documented and reported in the final trial report. Note that, where at least one hospital site within a cluster is eligible, the cluster will be regarded as eligible. Where multiple hospital sites are eligible within a cluster, the NCA may treat them as separate but they will be regarded as a single cluster for the purposes of randomisation.

8.3 Clinical audit case selection

The detailed process for selecting cases for inclusion in the clinical audit will be decided by the NCA BT Project Group for each topic and will be detailed in the ***NCA Audit Protocol*** and documented in the ***Trial Statistical Analysis Plan***.

As a minimum, this will include:

- Eligibility criteria for consecutive cases to be included / excluded from the audit
- Clear process to ensure the appropriate number of cases is audited at each site and a detailed process on how they are screened and selected.
- Data collection tools which have been pre-piloted.

9 Randomisation of NHS Trusts / Health Boards

The NCA will inform the CTRU, University of Leeds, when each baseline audit is complete and the audit database has been locked for analysis.

Trusts or Health Boards will be randomised by the CTRU using an automated randomisation system based at the CTRU. It will remain possible to participate in each topic until each baseline audit database has been locked.

Trusts or Health Boards that fulfil the eligibility criteria, have NHS Permissions to participate and have completed the baseline audit will be randomised on a 1:1:1:1 basis to receive one of four feedback interventions twice, once after each baseline audit.

A computer-generated minimisation programme incorporating a random element will be used to ensure intervention arms are balanced for the following cluster-level characteristics:

- Trust size. For each audit we will review the number of cases submitted to baseline audit per cluster and then break these down into tertiles (Large, Medium and Small); the final cutpoints used will then be reported. This data was provided by the NCA after the close of audit data collection.
- Regional Transfusion Committee (RTC): refer to Appendix C for details of the RTC

For the second randomisation to the haematology audit, an additional stratification factor will be used which will include the previous randomised allocation to the first trial.

Where Trusts or Health Boards merge following each randomisation they will continue to be regarded as separate and distinct clusters for intervention, data collection and analysis purposes. For clusters that merge between trials dependent upon review these will continue to be regarded as distinct clusters as well. This will be accounted for in a sensitivity analysis.

Following each randomisation, the CTRU will inform the NCA, the Chief Investigators and the intervention delivery team so that appropriate arrangements can be made to facilitate the development and the delivery of the feedback according to randomised allocation.

Other personnel involved in the trial will only be informed of intervention allocation if this is required to undertake their role associated with the trial. A log will be maintained of who is unblinded to what at specific points in the research process.

10 Feedback-related interventions

Detailed protocols specifying the intended delivery of each component of the trial interventions will be written by the Intervention Leads (including Simon Stanworth, Natalie Gould, Fabiana Lorencatto and Robbie Foy) and finalised following completion of Work Stream 1. The intervention protocols will not be disseminated to clinical audiences until the results of the two trials are in the public domain.

10.1 Usual feedback

Current practice is defined as the standard feedback delivered by the NCA following completion of an audit, targeting clinical teams within Trusts or Health Boards. Feedback is typically in the form of a written clinical audit report to hospital sites, a regional PowerPoint presentation and (often) action plan templates. The content of the written report varies, depending on the audit. How these clinical teams and organisations respond following receipt of the feedback is regarded as a consequence of the trial interventions – but is presently considered to be highly variable. We expect them to respond in the context of their clinical governance arrangements. No restrictions will be imposed on current practice or on Trusts or Health Boards undertaking additional development or training in the provision of feedback, with the exception that we will request that the staff who receive the feedback do not share with colleagues external to their own Trust or Health Board.

10.2 Enhanced feedback

The ‘enhanced’ feedback has been developed using the current evidence base for A&F [15] and behaviour change theory [16-17]. While its proposed effects are theoretically plausible, there is real uncertainty regarding whether this will translate into improved outcomes. Equipoise will be actively encouraged within the research team and the clinical community until the results of the trial are known.

Enhanced feedback documents: feedback documents with content written to specifically deliver behaviourally specified feedback and the relevant theoretically-consistent behaviour change techniques. These documents will be delivered as per usual practice by the NCA programme through written and graphic feedback presented in multiple feedback documents and presentations.

Post-feedback support: targeted dissemination of the feedback to relevant staff with discussion and agreement of action plans. The post-feedback support intervention comprises practical guidance for clinical teams on how to operationalise the process of responding to feedback, including materials for clinical teams to facilitate discussion and agreement of locally relevant goals and action plans based on feedback.

11 Definition of end of both trials

The end of trial is defined as the last clinical audit case collected at the time of follow-up of the Haematological audit.

12 Monitoring intervention adherence & fidelity

The AFFINITIE Programme includes a process evaluation to provide a detailed understanding of how the feedback interventions were delivered in practice and how staff responded to them. Specifically, the AFFINITIE process evaluation will be based on the Medical Research Council (MRC) guidance for conducting process evaluations of complex interventions [18]. This guidance recommends assessing: i) the extent to which interventions are implemented by the intervention providers and enacted by intervention recipients as intended (i.e. with fidelity); ii) potential contextual influences on intervention implementation (e.g. publication of new transfusion guidelines, levels of available resources, staff); and iii) the extent to which degree of fidelity mediates intervention outcome.[18]. Specifically, the assessment of fidelity in the AFFINITIE program will also be based on a fidelity framework proposed by the NIH Behaviour Change Consortium (BCC) [19-20] to investigate and report the extent to which the enhanced and standard feedback interventions were (i) designed, (ii) trained, (iii) delivered, (iv) received and (v) enacted as intended (i.e. with fidelity). Two linked process evaluations will be conducted alongside the two linked cluster randomised trials.

As part of the trial, the intervention developers and intervention providers will fully document their activities to provide a record of fidelity in terms of study design, provider training and intervention delivery (refer to section 13.5). This is necessary for monitoring the conduct of the trial but it will also be used to calculate estimates of the costs of the interventions and to inform analyses relating to mediators of the effect of randomised allocations on outcome.

Data collected from the Hospital Transfusion Teams for all clusters will be limited, including questionnaires to collect high-level data on contextual influences, the receipt and enactment of the interventions (e.g. confirmation of receipt of documentation and overall rating of the level of enactment of post-feedback support) (refer to section 13.5). These questionnaires will be sent to all hospitals approximately six months following intervention delivery.

Additional data will be collected for all Trusts randomised to receive Intervention 2 (i.e. enhanced post-feedback support). The post-feedback support intervention consists of two components: i) a web-based toolkit to support hospitals in planning their response to the feedback reports, including problem solving, goal setting, and action planning tools; and ii) telephone support sessions with a member(s) of the hospital transfusion team (e.g. transfusion practitioner) to promote engagement with, and facilitate use of, the web-based toolkit. In order to monitor the implementation of Intervention 2, all telephone support sessions will be audio-recorded to assess the extent to which the intervention providers delivered the telephone support with fidelity to the intervention manual. Informed verbal consent will be sought from the intervention recipient at the beginning of the call prior to commencing audio-recording; the telephone support session will not be audio-recorded if the intervention recipient does not provide informed consent. Quantitative web analytics data will also be collected on the extent to which intervention recipients engage with the web-based toolkit, including: number of log-ins, duration of log in, number of web pages viewed, and extent of completion of problem solving, action planning and goal-setting tools. This data will be collected automatically without additional input required from the intervention recipients. The web analytics data being collected will be clearly stated on the log-in page for the toolkit. Informed consent will be sought upon log-in to record this data, providing intervention recipients with the option to opt out of data collection.

All telephone support and web analytics data will be collected throughout the intervention delivery period, and cease upon collection of follow-up outcome data collection for each trial. At the point of data analysis, any identifying or personal information will be removed from the transcripts of the audio-recorded telephone support sessions and all web analytics data so that no individual clinical staff member or Trust may be identified directly from the data. Only AFFINITIE researchers will have access to the records and be able to re-identify the data (in the event of a participant requesting their data be removed from the programme). All data will be handled in accordance with the Data Protection Act 1998, and will be stored in secure locked cupboards, or on secure, password protected computers at City University. Data will be destroyed after a minimum of five years after the end of the trial, deleting all electronic files and shredding any documentation.

12.1 Process evaluation with sub-sample of hospitals

In addition to the above intervention adherence and fidelity assessments, a more detailed process evaluation will be conducted with a sub-sample of participating hospitals to explore in more detail how the interventions have been delivered, received and implemented. Specifically: i) context, ii) fidelity of receipt, and iii) fidelity of enactment will be investigated. ‘Context’ may refer to any factors external to the interventions being delivered, that may facilitate or hinder the attainment of target outcomes (e.g. organisational norms, motivation, attitudes, clinical guidelines, policy changes, extent of available resources).[18] Fidelity of receipt concerns the extent to which the two enhanced A&F interventions are understood and engaged with by the target intervention recipients (e.g. the hospital transfusion team). Fidelity of enactment concerns whether intervention recipients subsequently apply the intervention in their day-to-day clinical practice as intended (e.g. read and disseminate the feedback reports, implement developed action plans, respond to feedback recommendations)[19-20].

12.1.1 Design

Semi-structured qualitative interview study

12.1.2 Participants and recruitment

For each trial, once the interventions have been delivered, interviews will be conducted with up to three clinical staff members from three Trusts from each trial arm (i.e. 3 Trusts x 4 trial arms= 12 Trusts; 12 Trusts x 3 clinical staff= 36 participants in total per trial; 24 Trusts and 72 participants across both trials).

Trusts will be eligible to participate if they have been randomised to take part in the trial and have received an A&F intervention according to their trial arm allocation [i.e. i) standard reports + no enhanced post-feedback support; ii) enhanced reports+ no enhanced post-feedback support; iii) standard reports + enhanced post-feedback support; iv) enhanced reports + enhanced post-feedback support]. Trusts that have been involved in earlier interviews conducted as part of intervention development and piloting will be excluded, as they have been previously exposed to the enhanced intervention components and therefore were not randomised as part of the trial. Twelve Trusts will be randomly selected from those eligible to participate in Trial 1. In Trial 2, a new sample of twelve randomly selected Trusts will be sampled from those eligible hospitals taking part in Trial 2. To maximise the number

of different Trusts interviewed, Trusts that participated in the interview study for Trial 1 will be excluded from the randomised sample in Trial 2. Random selection will be performed by a CTRU statistician.

Clinical staff eligible to participate will include two members of the hospital transfusion team (e.g. transfusion practitioner, consultant haematologist), and a clinical staff member from the relevant specialty/topic being audited (e.g. Trial 1 is an audit of patient blood management in elective surgery, therefore anaesthetists or surgeons would be eligible). The clinical contact for the audit (i.e. typically a transfusion practitioner or consultant haematologist) at each of the eligible, randomly selected, Trusts will be contacted in writing (e.g. letter and/or email) with a study information sheet and invitation to participate in the interview study. The clinical contact will be asked to complete a consent form to confirm that their hospital is willing to participate in the interview study. Upon agreeing to take part, the research team will ask the clinical contact within each Trust to provide guidance as to whom to interview by assisting in the identification of other members of the hospital transfusion team and representatives from the relevant specialty. Any potential interview participants will be sent a recruitment message from one of the researchers, with an information sheet and consent form provided. Interviewees will be asked to provide individual consent before participating in the interviews, and will be able to decline participation if desired.

Participation in the interview study is entirely voluntary. Whether or not potentially eligible Trusts invited to participate in the interview studies agree to take part will not affect the delivery of the A&F interventions that the Trusts have been allocated to receive. If any Trusts decline to take part, a further sub-sample of Trusts will be randomly sampled until recruitment objectives are achieved for each trial arm.

12.1.3 Procedure

The semi-structured interview topic guide will be based on the Theoretical Domains Framework- an established psychological framework that has been applied in a range of clinical contexts, including transfusion medicine, to explore barriers and facilitators to behaviour and behaviour change [17]. The interviews will examine: i) contextual factors acting as barriers or enablers to the delivered A&F interventions being acted upon (e.g. extent of social support from colleagues, available resources), and contextual factors influencing transfusion practice more generally (e.g. publication of new NICE guidelines); ii) the extent to which intervention recipients understood and iii) enacted the interventions (e.g. how much of the feedback reports did the interviewee read? Did they understand the key findings/recommendations? Did they discuss the reports with colleagues? Did they log into the web toolkit? Did they implement their action?).

The interviews will last a maximum of one hour, and will be conducted at a time and location of convenience to the participants. Convenient times and dates to visit the hospital will be discussed between the researchers and interviewees. Participants will also be given the option to conduct the interviews via telephone if more convenient. All interviews will be audio-recorded and transcribed verbatim. Interviews will be fully anonymised at the point of analysis so that no individual clinical staff member or Trust may be identified directly from the data. Only AFFINITIE researchers will have access to the records and be able to re-identify the data (in the event of a participant requesting their data be removed from the

programme). All data will be handled in accordance with the Data Protection Act 1998, and will be stored in secure locked cupboards, or on secure, password protected computers at City University. Data will be destroyed after a minimum of five years after the end of the trial, deleting all electronic files and shredding any documentation.

12.1.4 Analysis

Interview responses will be analysed and synthesised across participants using the Theoretical Domains Framework as a coding framework for identifying and classifying beliefs regarding the different types of barriers/enablers to the implementation of the A&F interventions (e.g. ‘social pressure from my colleagues does (not) motivate me to change my practice in light of feedback;’ or ‘I was (not) able to readily identify the key findings from the feedback reports.’) Such data will aid the interpretation of observed trial outcomes and inform future refinements of the enhanced A&F interventions.

13 Data collection and data transfer

Data will be collected at the following time points:

- Cluster Screening (i.e. prior to inclusion in the NCA)
- Patient Registration (i.e. prior to the baseline and follow-up audit data collection)
- Cluster and Patient Baseline (e.g. the baseline audit data collection)
- Cluster and Patient Follow-up (e.g. the follow-up audit data collection)

Required data, collection time points and processes are summarised in Table 1 and subsequent sections below.

13.1 Summary of data collection

Table 1: Summary of Data Collected for each audit Topic

**Data (including who provides these data)**	**Timeline**			
	Screening	Registration	Baseline	Follow-Up
**Data regarding Trusts / Health Boards**				
Cluster level screening information (NCA → CTRU)	X			
Confirmation of cluster eligibility for the NCA (NCA → CTRU)	X			
NHS Permissions (Trust / Health Board →CTRU)	X			
Blood Stock Management (BSMS → CTRU→UCL)			X	X
SHOT Reportable Events (SHOT →CTRU→UCL)			X	X
Cluster Withdrawal (Trust / Health Board → CTRU)	Throughout the trial evaluation			
**Clinical Audit Data**				
Clinical Audit Cases (DCo → NCA→CTRU)		X	X	X
Organisation Survey (DCo →NCA→CTRU)			X	X
**Data regarding Data Collectors**				
Role of the data collector			X	X
**Data on Intervention Delivery**				
Intervention Fidelity (Design) (WS1→CTRU/WS3)				X & Y
Intervention Fidelity (Training) (ID→CTRU/WS3)				X & Y
Intervention Fidelity (Delivery) (IP→CTRU/WS3/Oxford)				X & Y
Intervention Fidelity (Receipt) (HTT→CTRU/WS3/Oxford)				X & Y
Intervention Fidelity (Enactment) (HTT→CTRU/WS3/Oxford)				X & Y
Contamination Events (NCA, ID, RTC→CTRU)				Z
Unblinding Events (DCo, NCA→CTRU)				Z
**Data on intervention costs**				
Resource inputs for audit data collection and submission (DCo → NCA→CTRU →UCL)			X	
Resource inputs for production and delivery of feedback documents (AL + NCA →CTRU → UCL)			X	
Resource inputs for 'post-feedback support' intervention (WS1→CTRU → UCL)			X & Y	

Data Collectors (DCo); SHOT=Serious Hazards of Transfusion database; BSMS=Blood Stocks Management Scheme database; AL=Audit Lead; HTT=Hospital Transfusion Team; ID=Intervention Developers; IP=Intervention Providers; WS1= AFFINITIE Work Stream 1; WS3=AFFINITIE Work Stream 3

X = Mainly quantitative data collected as part of Works Stream 2 by CTRU

Y = Mixed qualitative and quantitative data collected as part of Work Stream 3 process evaluation

Z = Mainly qualitative data collected as part of Work Stream 2 by wider Leeds team

13.2 Data regarding NHS Trusts / Health Boards

The data collected on NHS Trusts / Health boards will include the following:

13.2.1 Screening and confirmation of eligibility

The NCA will provide CTRU with screening data on all NHS Trusts / Health Boards in the UK and will include the following information:

- Name of NHS Trust / Health Board and individual hospitals
- Eligibility (i.e. perform procedures relevant to either audit topic) correct at the point of first approach by the NCA and interest in taking part in the audit
- Eligibility and interest in taking part in the cluster trial
- Trust and Health Board size (defined by volume of blood transfusions) to inform the stratification of the randomisation and the planned moderator analysis

Documented reasons for ineligibility or declining participation will be closely monitored by the trial team as part of a regular review of the recruitment process. Screening data will be collected and collated by the NCA and forwarded to the CTRU for inclusion in the trial dataset.

13.2.2 NHS Permissions

CTRU will liaise with the NHS Trusts / Health Boards to obtain the relevant NHS permission for participation in the evaluation of the feedback interventions. NHS Permissions will be used as evidence that the NHS Trust / Health Board consent to take part in the evaluation of the feedback interventions for one or both audit topics.

Reasons for not obtaining NHS Permissions will be obtained and documented in the trial dataset.

13.2.3 Blood Stocks Management Scheme

Blood Stock Management Scheme (BSMS) collects data in relation to blood stock and wastage management from hospitals in England.

CTRU will request from BSMS hospital level datasets which will cover the period 12 months before and 12 months after the feedback is provided to NHS Trusts / Health Boards. Linkage of individual patients between the two datasets will not be possible. The data will be provided in electronic format, via a secure file transfer system, and will include the following variables:

- Month (Oct 2014 to July 2017)
- Laboratory (ID and name)
- Hospital (ID and name)
- Trust or Health Board (DoH Trust ID and name)
- Hospital Profile (i.e. hospital-level descriptive data) (incl. electronic/manual cross-matching, cross-match reservation period, cell salvage availability, Regional Transfusion Committee)
- Blood Group (O, A, B, AB +/-)
- Gross issue data for RBC, platelets (and adult FFP)

- Net issue data for RBC, platelets (and adult FFP)
- Wastage data for RBC, platelets (and adult FFP)
- Transfused data for RBC, platelets (and adult FFP)

13.2.4 Serious Hazard of Transfusion

Serious Hazards of Transfusion (SHOT) is a UK wide haemovigilance scheme which collects anonymised data on adverse events and reactions associated with the transfusion of blood and blood components.

CTRU will request from SHOT fully anonymised patient level datasets which will cover the period 12 months prior to and 12 months after the feedback interventions begin. The data will be provided in electronic format, via a secure file transfer system, and will include the following variables:

- Unique Consecutive Incident ID
- Unique Consecutive Patient ID
- Speciality
- Hospital / Trust or Health Board
- Date of Transfusion
- Blood Components Transfused and/or Implicated Component(s)
- Source of Component (blood service donor, autologous, directed donation)
- Primary Diagnosis for the Component
- Transfusion Event (Adverse event, Pathological reaction, Transfusion Transmitted Infection, Pulmonary complication of transfusion)
- Type of Incident (Event, Near Miss, Right Blood Right Patient (RBRP))
- Date of Incident
- Status is: (i) pathological reaction which may not be preventable; (ii) probably or possibly preventable by improved practice and monitoring; or (iii) adverse event caused by error
- All datasets for Avoidable-, delayed- and under-transfusion (as per SHOT definitions).

These data will be used by the CTRU to derive baseline and outcome variables of interest for the trials at the randomised cluster-level. Once trust-level datasets have been linked, and prior to performing or reporting any analyses, all identifiers will be stripped and Trusts/Health Boards will only be identified by unique consecutive IDs.

For each incident type, data will be collected on investigations, treatments, support and outcomes so as to facilitate estimates of the costs and health outcomes of transfusion related adverse events for use as inputs into the health economic modelling. We will request data, already stripped of Trust and Health Board identifiers.

13.2.5 Contamination events

There is a potential for contamination of interventions, i.e. staff from sites receiving standard feedback being exposed to the intervention feedback. Contamination between intervention and standard arms may occur at up to six levels:

- Hospital Transfusion Team (e.g. Transfusion Practitioners) communicate with colleagues in other NHS Trusts / Health Boards as part of their role;

- NHS BT Patient Blood Management Practitioners – communication with colleagues in other NHS Trusts / Health Boards;
- Clinical Audit Leads;
- NCA writing group;
- Clinical staff – junior medical staff training and on rotation between different units and senior medical staff working across different sites;
- NCA Programme Manager and Statistician;
- The AFFINITIE Trial team.

We have assessed the respective risks and impacts of contamination at each level. We have taken several steps to minimise such risk. For example, education about these risks will be reiterated throughout the programme and we will check for, and monitor, any interactions within the main research programme team that may also lead to risks of contamination (e.g. to ensure that those involved in usual national audits only follow 'practice as usual' in preparing feedback for the trial control arms). We will use a combination of brief interviews, observations and diaries to gather data suggesting contamination and inform interpretation of trial findings.

13.3 Clinical Audit Data

To minimise the risk of detection bias, the NCA team will develop a *Data Collection Protocol* outlining how data are to be collected and will provide the data collectors with training in the clinical audit prior to the start of the audit. It is acknowledged that prior experience in clinical audit and the review of medical records may improve the quality of reporting. Availability of data collectors at the host organisation may also determine who can perform the audit. Therefore the NHS Trust / Health Board will be responsible for identifying the data collectors, with preference given for the people independent from the hospital transfusion team and the transfusion committees.

The NCA will ask NHS Trusts / Health Boards participating in the audit to collect data using supplied proformas and then key it into an online form or it will be keyed directly online depending on preference. Alternatively, NHS Trusts / Health Boards may transfer the completed proformas to the NCA who will then key it into the online form. Audit data will be retained in line with best practice.

The NCA will provide CTRU with four fully-anonymised patient level datasets, covering the baseline and follow-up audits for each of the two transfusion topics. The data will be provided in electronic format, via a secure file transfer system. The first dataset relating to the first audit topic will include the following variables:

All eligible cases during the reporting period:

- Consecutive unique patient ID
- Year of birth
- Sex
- Type of procedure
- Date of decision for surgery
- Patient status at time of audit (i.e. audited or notes not available)

All cases that can be audited:

- Investigations for anaemia
- Date of planned surgery
- Pre-operative treatment of anaemia
- Details of pre-operative transfusion (incl. pre-op Hb, volume of RBC, reason)
- Pre-operative acute coronary ischaemia
- Pre-operative medications (type, whether stopped)
- Date of actual surgery
- Details of intra-operative transfusion (incl. intra-op Hb, cell-salvage/allogeneic RBC, reason)
- Intra-operative medications (adjuvant treatments)
- Post-operative cell salvage (technique, volume)
- Post-operative complications
- Details of post-operative transfusions (incl. post-op Hb, volume RBC, reason)
- Date of discharge or death

This dataset will then be sent again for the follow-up audit.

For the second trial the dataset will include the following variables:

All eligible cases during the reporting period:

- Consecutive unique patient ID
- Year of birth
- Sex
- Haematological diagnosis
- Transfusion details (Red cell, platelet or both)
- Patient status at time of audit (i.e. audited or notes not available)

All cases that can be audited:

- Weight
- Haematology treatment details
- Participation in clinical trials
- Type of patient (inpatient or day patient)
- Details of Red Cell Transfusion (broad categorisation, clinical indication, pre-transfusion Hb, date, post-transfusion Hb)
- Amount of other red cell transfusions in January
- Details of Platelet Transfusion (broad categorisation, clinical indication, pre-transfusion Hb, date, post-transfusion Hb)
- Amount of other platelet transfusions in January

This dataset will then be sent again for the follow-up audit.

13.3.1 Organisational survey

The NCA will attempt to gather data at timelines corresponding to the baseline and follow-up on structural and resource factors which may influence local adherence to recommended practice (e.g. availability of cell salvage).

13.4 Data regarding data collectors

Data will be collected on the role of the data collector and will be categorised as following:

- Tier 1 = e.g. Hospital Transfusion Team / Committee;
- Tier 2 = e.g. Audit Department, Laboratory;
- Tier 3 = e.g. Senior Clinicians;
- Tier 4 = e.g. Junior Doctors;
- Tier 5 = e.g. Matrons / Nurses.

13.5 Data on intervention delivery

Data on intervention delivery will be collected by individuals from the central team or from members of the Hospital Transfusion Team (forms to be completed by the HTT are marked with an asterisk as indicated below). Data will be collected on case report forms / online questionnaires / in-built web analytics and forwarded to the CTRU:

Intervention Design:

- Intervention Design and Specification Checklist
- Intervention Provider Selection/Recruitment Checklist (this will include basic characteristics of providers: job title, training, relevant experience).
- Confounder and Implementation Setbacks Monitoring Form

Training:

- Training Delivery Log
- Training Competency Log
- Intervention Provider Selection/Recruitment Checklist

Delivery

- Delivery of Intervention Components Record
- Delivery of telephone support log
- Recipient Toolkit Access Log (Computer Generated; i.e. web analytics data of number of log ins, duration of log in, number of page views, completion of problem solving, goal setting, action planning tools)
- Non-Manual Specified Intervention Content Delivery Record

Receipt

- Intervention Comprehension and receipt Questionnaire*
- Receipt of NCA Documentation (Computer Generated; i.e. number of feedback report downloads)
- Brief Acceptability Questionnaire*

Enactment (Enhanced Interventions Only)

- Intervention Enactment Checklist*
- Enactment of web toolkit log (i.e. web analytics data)

13.6 Resource use and costs

To specifically inform the health economic analyses (see section 17), we will utilise the trial to gather data on the resources required for:

- Audit data collection and submission (time of hospital personnel recorded for a sub-sample). This will be facilitated by requesting data collectors at a small sample of Trusts / Health Boards to record (on the NCA proforma described in section 13.3 above) their job title and the time taken to extract the required audit data from patient case notes and hospital information systems. So as not to burden trial sites, the time taken to enter the data onto the NCA system will be estimated by having trial personnel enter data for a number of 'mock' audit cases.
- Development and delivery of standard and enhanced feedback documents (time of NCA personnel in designing and populating documents with audit data). The resources required to design and populate the standard and enhanced versions of the feedback documents will be identified during the trial by asking the NCA clinical audit leads and statistician to record the amount of time taken to perform each of these activities.
- Delivery of the post-feedback support (time of personnel delivering and receiving the post-feedback support session). Project team members delivering the 'post-feedback support' intervention will record the duration and attendance of personnel at the training session provided to each Hospital Transfusion Team. Data on whether hospitals act upon the guidance given and what resources they deploy will be collected as outlined in section 13.5.

13.7 Data Transfer

Data collected on the clinical audit cases and the organisational survey will be obtained via the NCA BT in line with standard agreed processes for conducting a NCA audit.

All electronic data transferred to the CTRU will be in the form of anonymised datasets as specified in the sections above. The CTRU will be able to link data on NHS Organisations across data sources but will not be able to do so for patients. Data Transfer Agreements (including details on the sender, recipient, content of transfer / general purpose of transfer and any data processing limitations) will be set-up between the following organisations prior to the transfer of any data:

- NCA BT and the CTRU
- SHOT and the CTRU
- BSMS and the CTRU
- CTRU and University of Oxford (for the health economics data)
- City University and CTRU (for the process evaluation data)

All data will be provided on the condition that it is stored, handled and processed in accordance with the principles of the 1998 Data Protection Act, the operation of the data transfer agreement and the Study Publication Policy. The rights for this data belong to the Study Sponsor and no processing, including further data transfer in whole or in part to a 3rd party, is permitted other than as stated in the data transfer agreements.

Other data collected (e.g. data collectors, intervention fidelity, contamination / unblind events will be reported to the CTRU on a paper CRF or electronically in a spreadsheet.

14 Withdrawal

It is possible for the NHS Trust / Health Boards to withdraw NHS Permission (i.e. consent) at any point during either trial after randomisation. NHS Trusts / Health Boards will be able to withdraw from the trial as a whole i.e. 'the intervention and trial data collection' or 'the intervention'. (Note: that this active withdrawal is distinctive from remaining in the allocated arm but not receiving the intervention or parts thereof for other reasons). NHS Trusts / Health Boards will withdraw consent by making contact with the CTRU. They will be asked to give a reason for their decision but are not required to do so if they prefer not to. Any data collected for that NHS Trust / Organisation up to the point of withdrawal would be used in the analysis. NHS Trusts / Organisations will be able to withdraw from one or both of the audit topics.

15 Data monitoring, data management & quality

15.1 Monitoring schedule

A Trial Monitoring Plan will be developed and agreed by the Trial Management Group (TMG) and Trial Steering Committee (TSC) based on the trial risk assessment. This Trial Monitoring Plan will be developed considering the scientific value of the research (including the potential risk associated with the implementation of the intervention and contamination which can, if not monitored and mitigated, affect the integrity and smooth running of this cluster randomised trial). This monitoring plan will detail the timing and content of reports to monitor trial conduct and implementation and adherence with the Consolidated Standards of Reporting Trials (CONSORT) - extension to cluster randomised trials [20].

The NCA will implement a data management plan to include ongoing checking of data quality and completeness during the clinical audit collections. Details on processes undertaken by the NCA to adhere to their standards for ensuring data quality in clinical audits will be followed. In brief, during the audit periods, the NCA team will monitor the cases audited at each site and will review data received to identify missing and illogical information. This will help maximise the number of cases reported, the completeness and quality of the data and will allow for monitoring the adherence to the list of cases selected for audit. As a minimum this will include:

- Assessing the data received to identify missing or erroneous data items and chasing this with the data collectors at site until they are confirmed as not available, or when the clinical audit closes.

- Checking for data input errors where data is transferred from paper to the online system at the NCA.
- Monitoring the number of audit cases started at each site and chasing the data collectors at site if progress is not as expected.

Data provided to the CTRU will be monitored for quality and completeness by the CTRU, using verification, validation and checking processes as appropriate. Data will be queried with the source where possible.

16 Analysis plan and statistical considerations

16.1 Endpoints

16.1.1 Primary endpoint

The primary outcome for each audit topic, measured at the patient level and taken from the NCA follow-up audit, is whether a transfusion is categorised as unnecessary or not (binary).

A clinical algorithm for determining if a transfusion is unnecessary or not will agreed upon by an independent panel of 2 clinicians and a statistician based on clinical relevance and baseline compliance. They will first be presented with a clinical description of candidate endpoints that could be suitable based on the current literature and the basis of the audit. Following this they will then be instructed to discuss the clinical relevance and merits of these endpoints and put forward a list of candidate endpoints they would like to investigate further. For these endpoints we will then present the panel, using a sample of baseline audit, summary information on baseline achievement of the endpoint and the sample size of the final candidate endpoints to make sure that the selected endpoint does not reduce the sample size below acceptable levels or we already have higher compliance than allowed for in sample size calculation. Following a final review of the final candidate endpoints accounting for both the clinical and statistical aspects the panel members will be asked to vote for their preferred outcome with the outcome that has the majority of votes being selected as primary endpoint for the trial. This will minimise the risk of detection bias. The final versions will be included in the Statistical Analysis Plan.

For the surgical audit, transfusion may occur pre-operatively, intra-operatively or post-operatively. There may also be multiple transfusion episodes after surgery but prior to discharge. As all patients will have had one or more transfusions over the entire operative period (14 days prior to surgery to 7 days following surgery), the primary outcome, agreed by the Outcome Review Panel, is whether any of the pre-operative and post-operative transfusions were unnecessary versus all pre-operative and post-operative transfusions being necessary (binary). The statistical algorithm given in the Statistical Analysis Plan will specify the statistical process needed to derive the primary outcome from the patient-level NCA audit. No clinical judgement will be required at a patient-level to categorise transfusions.

For the Haematological audit, there may be multiple transfusions for an individual within the auditing period. In such cases, that patient will be audited once only for one of those events.

A patient may also be transfused with both red cells and platelets in the audit period. In such cases, the patient may provide audit information for both the red cell transfusion and the platelet transfusion. The primary outcome is whether any of these transfusions were unnecessary versus all transfusions being necessary (binary). The statistical algorithm given in the Statistical Analysis Plan will specify the statistical process needed to derive the primary outcome from the patient-level NCA audit. No clinical judgement will be required at a patient-level to categorise transfusions.

16.1.2 Secondary endpoints

For the surgical audit *secondary outcomes* are:

1. Total volume of allogeneic RBC transfused (Units at trust-level, from BSMS summed over blood groups and clinical specialities, calculated from net-issue minus wastage)
2. Total volume of allogeneic RBC transfused (Units at patient-level, from NCA summed over pre-operative, intra-operative and post-operative periods);
3. Total number of incidents reported to SHOT (Count at trust-level, from SHOT summed over clinical specialities and events, near misses and "right blood right patient" incidents)
4. Number of definitely, probably or possibly preventable incidents reported to SHOT within clinical specialities targeted by the audit (Count at trust-level, from SHOT summed over events, near misses and "right blood right patient" incidents)

For the Haematology audit *secondary outcomes* are:

1. Total volume of allogeneic RBC transfused (Units at trust-level, from BSMS summed over blood groups and clinical specialities, calculated from net-issue minus wastage)
2. Total volume of allogeneic RBC transfused (Units at patient-level, from NCA)
3. Total volume of platelets transfused (Units at trust-level, from BSMS summed over clinical specialities, calculated from net-issue minus wastage)
4. Total volume of platelets transfused (Units at patient-level, from NCA)
5. Total number of incidents reported to SHOT (Count at trust-level, from SHOT summed over clinical specialities, and events, near misses and "right blood right patients incidents)
6. Number of definitely, probably or possibly preventable incidents reported to SHOT within clinical specialities targeted by the audit (Count at trust-level, from SHOT summed over events, near misses and "right blood right patient" incidents)

16.1.3 Tertiary endpoints

For the surgical audit, *supportive outcomes* are:

1. Pre-operative transfusion (Unnecessary/Grey/Necessary, from NCA)
2. Intra-operative transfusion (Unnecessary/Grey /Necessary, from NCA)
3. Post-operative transfusion (Unnecessary/Grey /Necessary, from NCA)
4. Individual NCA audit standard met (Yes/No, from NCA)
5. Proportion of NCA audit standards met (from NCA)
6. Total volume of RBC issued (Units at trust-level, from BSMS gross issue data)
7. Total volume of RBC transfused (Units at trust-level, from BSMS transfused data)
8. Pre-operative volume of allogeneic RBC transfused (Units at patient-level, from NCA)
9. Intra-operative volume of allogeneic RBC transfused (Units at patient-level, from NCA)
10. Post-operative volume of allogeneic RBC transfused (Units at patient-level, from NCA)

For the Haematology audit, *supportive outcomes* are:

1. Red blood cell transfusion alone (Unnecessary/Grey/Necessary, from NCA)
2. Platelet transfusion alone (Unnecessary/Grey/Necessary, from NCA)
3. Individual NCA audit standard met (Yes/No, from NCA)
4. Proportion of NCA standards met (from NCA)
5. Total volume of RBC issued (Units at trust-level, from BSMS gross issue data)
6. Total volume of RBC transfused (Units at trust-level, from BSMS transfused data)
7. Total volume of Platelets issued (Units at trust-level, from BSMS gross issue data)
8. Total volume of Platelets transfused (Units at trust-level, from BSMS transfused data)

For the surgical audit, patient-level *process variables* include:

1. Whether the planned surgery date equals the actual surgery date;
2. Volume of post-operative cell salvage transfused;
3. Hb level;
4. Length of post-operative hospital stay.

For the haematology audit, patient-level *process variables* include:

1. Whether the platelets transfused were HLA matched or not;
2. Hb level;
3. Platelet count.

For both the surgical and haematological audit, trust-level process data will be collected on:

1. Intervention Fidelity
2. Organisational structures and resources
3. Tier of Data Collectors

16.2 Sample size

The sample size calculation has been refined following the grant application in light of decisions regarding the feedback interventions and transfusion topics. For each topic, there are two principal comparisons of interest (1. enhanced feedback documents vs. usual documents; and 2. Post-feedback support vs. no post-feedback support), relating to the two main effects of the 2X2 factorial design. Assuming that there will be 20% unnecessary transfusions at follow-up for each topic, that the ICC will be 0.05 and that cluster sizes will vary from 17 to 68 with a mean of 45, we need 152 clusters to detect a minimally important reduction of 6% (i.e. to 14% unnecessary transfusions) in the presence of, at most, a small antagonistic statistical interaction (i.e. 10% or fewer unnecessary transfusions in the enhanced feedback documents and post-feedback support) with 80% power using logistic regression models, with a random-intercept for cluster, and a 2-sided 2.5% significance level, for each comparison within each model. This requires us to recruit from England, Wales, Scotland and Northern Ireland and allows 12/171 (7%) clusters to be ineligible and 95% of those eligible to consent and be randomised. If these assumptions do not all hold, the tables below illustrate the impact on the minimally important clinical difference the trial would be powered to detect.

Table 2: Sensitivity Analyses for ICC and recruitment assumptions fixing the power to detect the main effects at 80% & baseline proportion of unnecessary transfusions at 20%

ICC	150 Clusters	140 Clusters	130 Clusters
**0.05**	(20, 14, 10.0)	(20, 13.5, 9.8)	(20, 13.0, 9.5)
**0.06**	(20, 13, 9.5)	(20, 12.5, 9.3)	(20, 12.5, 8.9)

**0.07**	(20, 13, 9.0)	(20, 12.0, 8.8)	(20, 12.0, 8.4)
**0.08**	(20, 12, 8.5)	(20, 11.5, 8.3)	(20, 11.5, 7.9)
**0.09**	(20, 12, 8.0)	(20, 11.0, 7.8)	(20, 11.0, 7.4)
**0.10**	(20, 11, 7.8)	(20, 10.5, 7.5)	(20, 10.5, 7.0)

Note: The first number in brackets (i.e. 20) is the proportion of unnecessary transfusions in the standard/standard arm. The second number is the largest proportion of unnecessary transfusions we could detect with 80% power in either the enhanced/standard or standard/enhanced arms. The last number is the largest proportion of unnecessary transfusions we could have in the enhanced/enhanced arm to be able to detect the main effects with 80% power. The power to detect the interaction ranges from 5% to 25% under these scenarios.

Table 3: Sensitivity Analyses for ICC and recruitment assumptions fixing the power to detect the main effects at 80% & increasing the baseline proportion of unnecessary transfusions to 30% or 40%

ICC	150 Clusters	140 Clusters	130 Clusters
**0.05**	(30, 23.0, 18.1)	(30, 22.5, 17.7)	(30, 22.0, 17.3)
**0.06**	(30, 22.5, 17.3)	(30, 22.0, 17.0)	(30, 21.5, 16.5)
**0.07**	(30, 22.0, 16.7)	(30, 21.5, 16.2)	(30, 21.0, 15.8)
**0.08**	(30, 21.5, 16.0)	(30, 21.0, 15.6)	(30, 20.5, 15.1)
**0.09**	(30, 21.0, 15.4)	(30, 20.5, 15.0)	(30, 20.0, 14.5)
**0.10**	(30, 20.5, 14.9)	(30, 20.0, 14.4)	(30, 19.5, 13.9)
**ICC**	**150 Clusters**	**140 Clusters**	**130 Clusters**
**0.05**	(40, 32.0, 26.9)	(40, 31.5, 26.4)	(40, 31.0, 26.0)
**0.06**	(40, 31.5, 26.0)	(40, 31.0, 25.5)	(40, 30.5, 25.1)
**0.07**	(40, 31.0, 25.2)	(40, 30.5, 24.7)	(40, 30.0, 24.2)
**0.08**	(40, 30.5, 24.5)	(40, 30.0, 24.0)	(40, 29.5, 23.4)
**0.09**	(40, 30.0, 23.8)	(40, 29.5, 23.2)	(40, 29.0, 22.7)
**0.10**	(40, 29.5, 23.0)	(40, 29.0, 22.5)	(40, 28.5, 22.0)

Note: The first number in brackets is the proportion of unnecessary transfusions in the standard/standard arm. The second number is the largest proportion of unnecessary transfusions we could detect with 80% power in either the enhanced/standard or standard/enhanced arms. The last number is the largest proportion of unnecessary transfusions we could have in the enhanced/enhanced arm to be able to detect the main effects with 80% power. The power to detect the interaction ranges from 4% to 9% under these scenarios.

The worst case scenario is highlighted in grey. Under this, the minimum sample size would be required, that is 5940 patients to provide outcome data (i.e. 1485 patients across 132 clusters). The target sample size is 6840 patients to provide outcome data (i.e. 1710 patients and 38 clusters in each of four arms). The assumptions underlying the sample size calculation will be monitored throughout the project and any deviations reported to the oversight committees.

16.3 Statistical analysis

16.3.1 General considerations

Statistical analysis is the responsibility of the CTRU Statisticians. The analysis plan outlined in Section 18.3 will be reviewed, and a detailed Statistical Analysis Plan (SAP) approved, before any formal analysis is undertaken. The SAP will be drafted in accordance with CTRU standard operating procedures (SOPs) and will be finalised and agreed by the trial and supervising statisticians, the Chief Investigator and other appropriate members of the research team. Any changes to the final approved SAP, and reasons for change, will be documented.

The proportion of missing data is anticipated to be non-trivial, making the handling of missing data an important issue for the analysis. Mechanisms for missing data on key variables will

be explored, with the proportion of missing data to be compared between intervention arms, and a multiple imputation model built covering the main analyses. As a sizeable proportion of patients are expected to be missing from the main analyses and the missing data predictable by known variables, the principal method for handling this will be multiple imputation under a Missing at Random (MAR) assumption. Sensitivity analyses will be carried out to assess the impact of the choice of imputation model and of assuming data are Missing Not at Random (MNAR), as appropriate.

As the primary clinical effectiveness analysis for each topic has a single primary outcome but two main treatment effects, 2-sided 2.5% significance levels will be used for these contrasts. Where results are subsequently combined when interpreting the treatment effect (e.g. across secondary outcomes), full consideration will be given to the family-wise error rate and further adjustments made for multiplicity, as appropriate.

Cluster randomisation of trusts/health boards rather than individual-randomisation of patients to interventions imposes recruitment-related clustering effects whereby patient outcomes are expected to be correlated. As the impact of cluster randomisation is expected to be equal across arms, the principal method for handling clustering will be to fit a multilevel model that constrains the cluster and patient level variances to be equal across arms, that is, a random intercept model. Sources of treatment-related clustering have been considered (e.g. provider and decision-maker) but there is only one provider (i.e. NCA) and it is not possible to collect linked data on the health professionals making transfusion decisions. As such, we recognise it as an unavoidable limitation that we will ignore this in the analysis.

Data distributions will be summarised, cluster and patient-level CONSORT diagrams will be generated and characteristics of clusters and patients at baseline and follow-up will also be summarised by intervention arm.

16.3.2 Frequency of analysis

No interim analyses are planned. The two audit topics will be regarded as two trials, but with a single final analysis planned when all follow-up data from both topics has been databased, cleaned and locked. Screening and baseline data may be analysed separately.

16.3.3 Analysis populations

The primary analyses will be carried out on an intention to treat basis, utilising all available follow-up data from all consecutive patients and imputing unavailable follow-up data, comparing allocated interventions. A complier average causal effect (CACE) analysis, comparing treatments received, will be considered if more than 10% of trusts or health boards do not implement the intervention as intended. This decision will be made without reference to the effectiveness data.

16.3.4 Primary endpoint analysis

For each topic independently, the patient-level binary primary outcome of unnecessary transfusions 12 months following randomisation will be analysed using logistic regression, with a random intercept for trust/health board, adjusting for design factors (that is, trust size, regional transfusion committee, and trust-level proportion of unnecessary transfusions at baseline), with contrasts for (1) enhanced vs. standard documents, (2) post-feedback support vs. no post-feedback support, and (3) the interaction between (1) and (2).

Unadjusted and adjusted intra-cluster correlations (ICCs) and corresponding 95% confidence intervals will be presented. We will report the estimates and two-sided 97.5% confidence intervals for the two main effects from this model.

In addition to sensitivity analyses relating to assumptions about the missing data mechanism we will also analyse the supportive outcomes that include a grey-area category using ordinal logistic regressions and look for consistency in the size, direction and interpretation of effect.

Following the analysis of each topic independently, a further analysis will be conducted using data from both audit topics. This analysis uses a bivariate patient-level binary primary endpoint outcome of unnecessary transfusions 12 months following randomisation and will analysed using multivariate logistic regression, with a random intercept for trust/health board and will be adjusted for the same design factors as above and additionally controlled for audit topic. The model will then include contrasts for (1) enhanced vs. standard documents, (2) post-feedback support vs. no post-feedback support and (3) the interaction between (1) and (2). We will then fit all possible interactions between (1), (2), (3) and audit topic; we do this in order to get unbiased estimators for the general effects of interventions accounting for possible interactive effects between the two trials on the intervention effects. Unadjusted and adjusted intra-cluster correlations (ICCs) and corresponding 95% confidence intervals will be presented. We will report the estimates and two-sided 97.5% confidence intervals for the two main effects from this model and also for the interactions between (1) and (2) and audit topic.

The same sensitivity analyses will be run on this model as with the separate models.

16.3.5 Secondary endpoint analyses

The patient-level secondary endpoints for both trials covering volume transfused (both RBC and platelets) will be analysed using a Poisson random intercept regression model, with the same contrasts and adjusting for the same covariates as in the primary endpoint analysis. The trust-level secondary endpoints (volume transfused, number of SHOT-reportable incidents, and number of definitely, probably or possibly preventable incidents reported to SHOT within clinical specialities targeted by the audit) will be analysed using cluster-level analyses recognising the outcomes are all counts, again with the same contrasts and adjusting for the same covariates as in the primary endpoint analysis

We will additionally analyse the supportive outcomes relating to the volume of transfused. The first two will act as sensitivity analyses for the trust-level analysis, indicating whether the size, direction and interpretation of the effect depend on the type of BSMS data summarised. The second three will act as a sensitivity analysis for the patient-level analysis, indicating whether the size, direction and interpretation of the effect depend on the stage in the operative process.

16.3.6 Further secondary analyses

Exploratory analyses will be conducted investigating mediators (e.g. fidelity: delivery, receipt and enactment) and moderators (e.g. trust size) of the main effects of the two feedback interventions in the surgical and haematological audits.

A number of exploratory sub-group analyses are planned which will be specified in detail in the SAP. These will include trust/health board and patient level factors such as trust size, TP involvement, surgical procedure and Haematological diagnosis.

Sensitivity analysis will be undertaken to investigate how contamination events, such as merging hospital trusts, might affect the size and direction of primary outcome measure.

Full details of all secondary analyses will be given in the Statistical Analysis Plan.

17 Safety

The programme of research aims to improve adherence to recommendations, and therefore the interests of patients will be safe-guarded by the normal duties of care. Our interventions will comprise different ways of providing feedback to clinical teams on clinical performance and will be embedded within an existing national clinical audit programme. These interventions themselves will therefore not be directed at individual patients but we are assessing the indirect (or downstream) impact on patient care to determine the effectiveness of the interventions and thus inform future NHS quality assurance activities. A main patient benefit (and outcome) is measured by reduced exposure to the risks of unnecessary transfusions - we are not exploring the risk of under-transfusions.

Anyone delivering the interventions and in the trial team will have the opportunity to highlight any issues of concern with the trial team for further consideration. This will include escalation to the TSC and notifying the individual NHS Organisations in line with appropriate information and clinical research governance approval procedures.

18 Economic evaluation

18.1 Design

Two cost-effectiveness analyses (one for each trial) will be conducted using decision analytic modelling. Both analyses will be conducted from the perspective of the NHS. To help inform decision making around the future implementation of A&F, the analysis requires a direct comparison of the costs and effects of all four A&F options (conventional A&F, enhanced content only, post-feedback support only, and combined enhanced content and post-feedback support) so as to determine which is likely to be the most cost-effective alternative and offer the best value for money. These works will draw on a variety of data collected specifically during the trial (see section 13 above and section 18.2 below), together with data generated elsewhere within and beyond the AFFINITIE Programme.

18.2 Methods

Trial-based economic evaluation is not feasible in this instance as no unique set of patients is identified at the start of the trial and followed until study completion. Health economic modelling is therefore required to simulate the costs and outcomes associated with the developed enhancements (separately and combined) as compared with current practice. Two models will be needed, one for patient blood management in surgery and one for haematology as the interventions will target these different transfusion topics and because the costs and adverse event profiles also differ between these components. The models will

be developed jointly by the CI and the project economist following a literature review of previously published models of transfusion, and in accordance with good practice guidelines for decision modelling. A number of transfusion specialists/haematologists external to the project will be asked to comment on the proposed model structure.

The models will combine decision tree and Markov approaches and will be structured so as to simulate the main hypothesised costs and effects. These include the costs associated with delivering standard and enhanced feedback interventions, the potential reduction in unnecessary transfusions and associated adverse events with their costs and consequences, as well as any change in clinical practice occurring to facilitate the reduction in transfusion e.g. for the surgery audit topic this might be better management of pre-surgery anaemia, use of tranexamic acid, or intra-operative cell salvage, all of which have associated costs which must be considered.

As detailed in section 13.6 above, data will be collected on a small sample of the clinical audit proformas used in the trials on time taken by hospital staff to identify and extract the required audit data during the trials. The time taken to enter these data onto the NCA on-line system will be estimated by members of the research team performing the data entry for a sample of 'mock' audit cases. These data will allow costs of the current NCA audit process to be quantified and will provide data for costing any new re-audits implemented at sites attempting to change behaviour following receipt of the post-feedback support). The resources required to design and populate the standard and enhanced versions of the feedback documents will be identified by asking the NCA clinical audit leads and statistician to record the amount of time required to perform such activities. Project team members delivering the 'post-feedback support' intervention will record the duration and attendance of personnel at the training session provided to each Hospital Transfusion Team. Data on whether hospitals then act upon the guidance given will be collected by a subsequent AFFINITIE work stream (work stream 3). The trials will also provide data on the current rate of unnecessary transfusions and the relative effectiveness of the interventions in reducing this rate for populating the model. Data will also be available via the trials on the volumes of blood components transfused to each audit case so as to estimate the mean number of product units per unnecessary transfusion. The organisational survey conducted at baseline and follow-up will include information on the components of patient blood management conducted at hospitals before and after the audit and will allow identification and costing of increased uptake of patient blood management policies in each trial arm. A combination of data from the published literature, expert clinical opinion, the UK haemovigilance system (Serious Hazards of Transfusion (SHOT)) and the Blood Stocks Management Scheme (BSMS), will be used to estimate the probability, cost, HRQoL, and survival impact of transfusion-related adverse events.

18.3 Analysis

A lifetime horizon will be used for the analysis and model results for each intervention will be reported as mean discounted costs and a series of outcomes (for example adverse events, life years, and quality adjusted life years). Incremental analyses will compare the costs and effects of each of the interventions versus standard A&F. Structural uncertainty in the model will be examined using sensitivity analysis and the model's internal consistency assessed using extreme value sensitivity analysis. Parameters will be entered into the model as

distributions and probabilistic sensitivity analysis used to assess the impact on the cost-effectiveness results of joint parameter uncertainty.

Cost effectiveness acceptability curves will identify the intervention most likely to be cost-effective for different values of willingness to pay for additional health gain. We shall liaise with the National Comparative Audit Team, SHOT, and NHSBT to ascertain whether any independent data are available to facilitate the external validation of the model.

The general methodology utilised to perform the cost-effectiveness analysis of the A&F interventions here will be described within a health economic analysis plan and will feed into the bank of resources planned for the knowledge exchange phase of the programme.

19 Clinical governance issues

Audit and feedback is a widely used quality improvement method which seeks to improve patient care and outcomes through careful review of health care performance against explicit standards. The aim of this trial is to evaluate different enhanced feedback interventions, targeted at individuals, teams, or services, to improve the impact on patient care. Therefore, the interests of the patients will be guarded by the normal duties of care.

Members of the NCA will feedback any issues to the individual NHS Organisations in line with appropriate information and clinical research governance requirements, as per usual practice in national audits.

Members of the central trial team who are involved in the delivering of the intervention will have the opportunity to highlight any issues of concern with the Trial Management Group, the Trial Steering Committee and to individual NHS Organisations in line with appropriate information and clinical research governance approval procedures.

20 Quality assurance & ethical considerations

20.1 Quality assurance

The study will be conducted in accordance with the principles of Good Clinical Practice (GCP) in clinical trials, as applicable under UK regulations, the NHS Research Governance Framework (RGF) and through adherence to CTRU Standard Operating Procedures (SOPs).

20.2 Serious breaches

Investigators are required promptly to notify the CTRU of a serious breach (as defined in the latest version of the National Research Ethics Service (NRES) SOP).

A ‘serious breach’ is defined as a breach of the protocol or of the conditions or principles of GCP (or equivalent standards for conduct of non-Clinical Trial of Investigational Medicinal Products (CTIMPs) which is likely to affect to a significant degree the safety or physical or mental integrity of the trial subjects, or the scientific value of the research.

Any unintentional disclosure of named patient or professional will be reported to NCA and the Trial Steering Committee and site concerned and will be handled in accordance with relevant Guidance.

In the event of doubt or for further information, the Investigator should contact the Trial Co-ordinator at the CTRU.

20.3 Ethical considerations

AFFINITIE will use an existing, established NCA as a platform to evaluate feedback interventions. The NHS BT NCA has previously performed national audits in approximately 98% of English Trusts, the majority of the Health Boards in Wales, ~50% of the Health Boards in Scotland and 100% of the Health and Social Care Trusts in Northern Ireland.

The NCA operates within robust information governance arrangements which are provided to the site at recruitment. Requirements for clinical audit are outside the remit of this trial protocol and will be followed in line with local practice for Clinical Audit oversight.

In line with the NCA standard practice, local hospitals will hold “linkage records” for patients and health professionals to facilitate the clinical audit data collection. The data entered online to the trial database will be unlinked and anonymised, and we will therefore not require individual consent from patients or health professionals. The NCA will ensure that robust processes are in place for the transfer of clinical audit data in line with best practice.

Ethical approval is not sought for the conduct of the clinical audit (with regards to design, data collection or analysis) of the audit as these are covered by local governance requirements. The interests of patients will be guarded by the normal duties of care. We will follow appropriate information and clinical research governance approval procedures.

The targets of the interventions are NHS professionals who are routinely involved in cascade of the feedback following a clinical audit; specifically:

- Hospital Transfusion Teams, Hospital Transfusion Committees
- Audit Departments and laboratories
- Senior clinicians / junior doctors
- Matrons / nurses
- Governance and Divisional Managers

The study will be submitted to and approved by a main Research Ethics Committee (REC) and will receive management approval for each participating NHS Trust/ prior to randomisation. The CTRU will provide the Main REC with a copy of the final protocol and all other relevant study documentation.

As this is a cluster trial, consent for participation in the trial will be obtained at the organisational level (i.e. by the NHS Trust / Health Board). Individual consent from the NHS Professionals in receipt of the feedback will not be obtained.

We will discuss any identified potential serious concerns about clinical practice with the NCA, Trial Steering Committee and relevant Trust or Health Board.

21 Confidentiality

Local clinical audit data will be stored and retained securely in accordance with the host organisations procedures.

All paper and electronic information collected by the NCA and the CTRU during the course of the project will be kept strictly confidential and will be retained and stored securely.

The project will comply with all aspects of the Data Protection Act 1998 and operationally this will include:

- Ensuring clinical audit data received by the NCA is anonymised and that only the instructed identifiers are present before sending to CTRU.
- Ensuring all data transferred to the CTRU is stored, handled and processed in accordance with the principles of the 1998 Data Protection Act, the operation of a Data Transfer Agreement, study specific guidance and the Study Publication Policy

22 Archiving

In accordance with the Department of Health's *Records Management NHS Code of Practice,* clinical audit records must be retained for securely for a minimum of 5 years after the clinical audit has completed. Arrangements for confidential destruction will be in line with the host organisation.

In accordance with the Sponsors policy, data collected for the purposes of the AFFINITIE trial evaluation will be securely archived for a minimum of 5 years after completion of the trial. Arrangements for confidential destruction will then be made.

No records may be destroyed without first obtaining written permission from the Sponsor.

23 Statement of indemnity

This study is sponsored by the NHS Blood and Transplant (NHS BT) and the NHS indemnity scheme will apply to meet the potential legal liability of the sponsor for negligent harm caused harm to participants arising from the management of the research.

Participants are NHS staff and indemnity to meet the potential legal liability of investigators/collaborators arising from harm to participants in the conduct of the research will be provided through the NHS schemes or through professional indemnity.

24 Study Organisational Structure

The general responsibilities are outlined below:

24.1 Sponsor

The Sponsor (NHSBT) is the organisation that takes responsibility for arrangements to initiate, manage, monitor and finance the trial. The Chief Investigator is employed by the NHSBT.

24.2 Chief Investigator

As defined by the NHS RGF, the Chief Investigator is responsible for the design, management and reporting of this study, the whole research programme and its constituent parts.

This trial will use an existing national audit platform, the National Comparative Audit, to evaluate audit and different types of feedback with the aim to reduce unnecessary transfusions. The background of the trial is standard practice and any intervention above standard practice will be delivered by a central trial team. The Chief Investigator will be accountable for the delivery of the feedback (intervention) and a Principal Investigator at each participating NHS Trust is not required. The supporting rationale for this is as follows:

- The target of the intervention are the staff employed by the NHS Trust. The role of a local Principal Investigator in this trial could draw additional attention to the trial and hence act as an unintended influence on local transfusion practice, thereby diluting any observed effect size of the interventions. It will not be possible to quantify the effect of this role which could undermine the scientific integrity of the trial.
- The intervention under investigation will be delivered by Intervention Providers who are part of a central team. The enactment following receipt of the intervention is part of the evaluation research and needs to follow real life as far as possible.

24.3 Clinical Trials Research Unit (CTRU)

The CTRU are responsible for the day to day conduct of the trial in accordance with the Research Governance Framework, where applicable, MRC GCP standards and the principles of CTRU SOPs. This includes randomisation design and implementation, database development and provision, protocol development, data collection, study design, monitoring schedule and statistical analysis and reporting. In addition, the CTRU will support main REC and Research and Development submissions, and site set-up and on-going management including non-clinical training, monitoring reports and promotion of the study.

The CTRU will be responsible for the trial database administrative functions and data management as agreed in the Trial Monitoring Plan and all statistical analyses.

24.4 Health Economists

The Health Economics collaborators will assist the TMG with development of the protocol and will be responsible for the selection and/or design of the economic data collection items, collation of unit costs, and the conduct, interpretation and writing up of the economic evaluations.

24.5 Staff responsible for Intervention Delivery

Staff from NHSBT and other NHS staff are already routinely involved in the delivery of national comparative audits. The NHSBT staff will advise on and support intervention development, site recruitment and data collection. The research staff from WS1 responsible for intervention development will be involved in training for and advising on intervention delivery. Existing NHS staff will be involved in the receipt and enactment of the intervention at regional or hospital levels in accordance with the training and guidance provided.

24.6 Audit Leads

Each of the Audit topics will be assigned two Consultants who will take on the role of Audit lead who will be part of the NCA BT Audit Group and will work with the NCA Programme Manager to develop and pilot the audit tool, develop the analysis plan and will develop the feedback documents in accordance with the intervention protocol.

24.7 Data Chasers

Data chasers will be employed by the NHS BT and will work with the NCA Programme Manager to implement the Data Management Plan as outlined in section 13.1.

24.8 Data Collectors

Data collectors will be the NHS staff who will complete the clinical audit on behalf of the local NHS Trust / Health Board. The NHS Trust / Health board will select who performs the data collection based on familiarity with the audit topic and ability to extract accurate data from hospital systems and records. Data Collectors will complete the audit tools for all retrievable cases registered and listed by the NCA

25 Operational Structure

25.1 Trial Steering Committee (TSC)

The Trial Steering Committee, with an independent Chair, will provide overall supervision of the trial, in particular trial progress, adherence to protocol, and consideration of new information. It will include an Independent Chair, not less than two other independent members, including a statistician. The Chief Investigator and other members of the TMG will attend the TSC meetings and present and report progress. The TSC will operate in line with the agreed Terms of Reference. TSC meetings will take place on the same dates as the AFFINITIE Programme Steering Committees.

25.2 Trial Management Group (TMG)

The TMG, comprising the Chief Investigator, CTRU team and members of the Programme Management Group will be assigned responsibility for the set-up, on-going management, promotion of the trial, and for the interpretation of results. Specifically the TMG will be responsible for (i) protocol completion, (ii) data collection requirements, (iii) obtaining approval from the main REC and supporting applications for NHS Permissions (iv) completing cost estimates and project initiation, (vi) facilitating the TSC, (vii) monitoring of site conduct (vii) interpretation of results and contribution to publications. The TMG will operate in line with the agreed Terms of Reference.

26 Publication policy

26.1 Principles of Authorship

The following principles of authorship are from editorial publications in leading journals [21,22] and are in accordance with the rules of the International Committee of Medical

Journal Editors (http://www.icmje.org/ethical_1author.html). These principles apply to any AFFINITIE outputs including papers and presentations.

26.1.1 Group authorship

- Group authorship will be appropriate when the intellectual work underpinning a publication 'has been carried out by a group, and no one person can be identified as having substantially greater responsibility for its contents than others' [21]. In such cases the authorship will be presented by the collective title – "The AFFINITIE Programme" and the article should carry a footnote of the names of the people (and their institutions) represented by the corporate title
- In some situations one or more authors may take responsibility for drafting the paper but all group members qualify as authors; in this case, this should be recognised using the byline 'Jane Doe ***and*** The AFFINITIE Programme' [22]
- Group authorship may also be appropriate for publications where one or more authors take full responsibility for the writing. Other group members are not authors but are listed in the acknowledgement (byline would read 'Jane Doe ***for*** *t*he AFFINITIE Study Group') [23].

26.1.2 Individual authorship

Other papers, such as describing satellite studies, will have individual authorship. To qualify for authorship an individual must fulfil the following criteria [21]:

i. Each author should have participated sufficiently in the work represented by the article to take public responsibility for the content.
ii. Participation must include three steps:
    - conception or design of the work represented by the article, and/or acquisition of data, and/or analysis and interpretation of the data; AND
    - drafting the article or revising it critically for important intellectual content; AND
    - final approval of the version to be published.

Participation in the collection of data is insufficient by itself. Persons who have contributed intellectually to the article, but whose contributions do not justify authorship, will be acknowledged and their contribution described.

26.1.3 Determining authorship and order of authors

- Tentative decisions on authorship should be made as soon as possible [21]. These should be agreed by the Chief Investigator. Any difficulties or disagreements will be resolved by the AFFINITIE steering group.
- Order of authors may be drafted at the proposal stage and confirmed when author contributions are clear. The first-named author takes responsibility for the processes of writing as described below; the last-named ("senior") author takes responsibility for the intellectual content of the paper; other authors are named in order of their contribution. Where contributions are equal, the group chooses one of the following:
    - Alphabetical order
    - Spread of author order across multiple papers to ensure equity, given overall contributions to the study

- Most journals require a statement of author contributions; this statement should be drafted by the lead author in consultation with the CI and approved by each author.

26.2 Authorship for Publications Arising from AFFINITIE

26.2.1 Operationalising authorship rules

We envisage two types of report (including conference presentations) arising from "The AFFINITIE Programme" and its associated projects:

i. *Reports of work arising from the "*AFFINITIE Programme*"* - If all grant-holders and research staff fulfil authorship rules, group authorship should be used under the collective title of "The AFFINITIE Programme"; if one or more individuals have made a significant contribution above and beyond other group members but where all group members fulfil authorship rules, authorship will be attributed to 'Jane Doe and "The AFFINITIE Programme".

ii. *Reports of individual studies and subsidiary projects* - Authorship should be guided by the authorship rules outlined in Section 26.1 above. Grant-holders and research staff not directly associated with the specific project should only be included as authors if they fulfil the authorship criteria. Grant-holders and research staff who have made a contribution to the project but do not fulfil authorship criteria should be recognised in the Acknowledgement section. The role of "The AFFINITIE Programme" in the development and support of the project should be recognised in the Acknowledgement section. The lead author should be responsible for ratifying authorship with the relevant AFFINITIE steering group.

For reports which specifically arise from "The AFFINITIE Programme" but where all members do not fulfil authorship rules (for example, specialist sub-study publications), authorship should be attributed to "Jane Doe for 'The AFFINITIE Programme'". If individual members of the group are dissatisfied by a decision, they can appeal to the relevant AFFINITIE steering group for reconciliation.

26.3 Quality assurance

Ensuring quality is essential to the good name of the AFFINITIE Programme. For reports of individual projects, internal peer review among relevant members of the AFFINITIE Programme Management Group is a requirement prior to submission of papers. All reports of work arising from "The AFFINITIE Programme" including conference abstracts should be peer reviewed by the AFFINITIE Programme Management Group.

The internal peer review for reports of work arising from "The AFFINITIE Programme" is mandatory and submission may be delayed or vetoed if there are serious concerns about the scientific quality of the report. .

The AFFINITIE steering group undertakes to respond to submission of articles for peer review following submission within two weeks of manuscript circulation.

26.4 Data source

Data from the CTRU database in Leeds must be used for data analyses for all abstracts and publications relating to the questions posed within the trial protocol. Furthermore, the statistical team at the CTRU must perform all such analyses. If any additional analyses outside the remit of the protocol are to be performed, the statistical team at the CTRU should be involved if it involves data held on the CTRU databases.

Data from the CTRU database in Leeds will be transferred to the Health Economic lead at the University of Oxford in accordance with the Health Economics Analysis plan and the relevant Data Transfer Agreement.

26.5 Data release

To maintain the scientific integrity of the trial, data will not be released prior to the first publication of the results of the study, either for trial publication or oral presentation purposes, without the permission of the TSC or the Chief Investigator.

The TSC will agree a publication plan and must be consulted prior to release or publication of any trial data.

Individual collaborators must not publish data concerning their participants which is directly relevant to the questions posed in the trial until the main results of the trial have been published. Local collaborators may not have access to trial data until after publication of the main trial results.

26.6 NIHR programme grant requirements

The NIHR must be notified prior to any publication (whether in oral, written or other form). A draft copy of any proposed publication must be sent to NIHR at the same time as submission for publication or at least 28 days before the date intended for publication, whichever is earlier.

All publications must acknowledge NIHR financial support and carry a disclaimer as directed by the NIHR. In the absence of specific direction, disclaimers should read: “This [poster / presentation / paper] presents independent research funded by the National Institute for Health Research (NIHR) under its Programme Grants for Applied Research Programme (Grant Reference Number RP-PG-1210-12010).” If the abstract and/or subsequent output is presenting research findings the following disclaimer should be included: “The views expressed are those of the author(s) and not necessarily those of the NHS, the NIHR or the Department of Health.”

27 References

1. Serious Hazards of Transfusion (SHOT) Steering Group. Knowles S and Cohen H (Eds) The2010 Annual SHOT Report (2011).

2. Stainsby D, Jones H, Asher D, Atterbury C, Boncinelli A, Brant L, Chapman CE, Davison K, Gerrard R, Gray A, Knowles S, Love EM, Milkins C, McClelland DB, Norfolk DR, Soldan K, Taylor C, Revill J, Williamson LM, Cohen H; SHOT Steering Group: Serious hazards of transfusion: a decade of hemovigilance in the UK. Transfus Med Rev 2006, 20:237-282.

3. Carless PA, Henry DA, Carson JL, Hebert PP, McClelland B, Ker K. Transfusion thresholds and other strategies for guiding allogeneic red blood cell transfusion. Cochrane Database of Systematic Reviews 2010, Issue 10. Art. No.: CD002042. DOI: 10.1002/14651858.CD002042.pub2

4. Carson JL, Kuriyan M. What should trigger a transfusion? Transfusion. 2010 Oct;50(10):2073-5

5. Stanworth SJ, Brunskill S, Hyde CJ, McClelland DBL, Murphy MF: Is fresh frozen plasma clinically effective? A systematic review of randomized controlled trials. Br J Haematol 2004a,126:139-152

6. Segal JB, Dzik WH. Paucity of studies to support that abnormal coagulation test resultspredict bleeding in the setting of invasive procedures: an evidence-based review. Transfusion. 2005 Sep;45(9):1413-25.

7. Stanworth S, Hyde C, Heddle N, Rebulla P, Brunskill S, Murphy MF. Prophylactic platelet transfusion for haemorrhage after chemotherapy and stem cell transplantation. Cochrane Database of Systematic Reviews 2004b, Issue 4. Art. No.: CD004269. DOI: 10.1002/14651858.CD004269.pub2; Update 2011 (submitted under review)

8. Stanworth SJ, Grant-Casey J, Lowe D, Laffan M, New H, Murphy MF, Allard S. The use of fresh frozen plasma in England: high levels of inappropriate use in adults and children. Transfusion 2010 Aug 27.

9. Estcourt L. National comparative audit of platelet transfusions, 2010. Key Findings of the audit with regard to the inappropriate use of platelet transfusions. http://hospital.blood.co.uk/library/pdf/PlateletRe-audit-Key_Findings_2010.pdf.

10. Wilson K, MacDougall L, Fergusson D, Graham I, Tinmouth A, Hébert PC. The effectiveness of interventions to reduce physician's levels of inappropriate transfusion: what can be learned from a systematic review of the literature. Transfusion, 2002(42): 1224-9.

11. Tinmouth A, Macdougall L, Fergusson D, Amin M, Graham ID, Hebert PC, Wilson K.., Reducing the amount of blood transfused: a systematic review of behavioral interventions to change physicians' transfusion practices. Archives of Internal Medicine, 2005. 165(8): 845- 852.

12. Born JA. Understanding the determinants of blood use in orthopaedics: a review of the literature and a theory based exploration of the factors influencing the decision to transfuse. MSc (Epidemiology) thesis. University of Ottawa. 2010.

13. Natalie J Gould, Fabiana Lorencatto, Simon J Stanworth, Susan Michie, Maria E Prior, Liz Glidewell, Jeremy M Grimshaw, and Jill J Francis Application of theory to enhance audit and feedback interventions to increase the uptake of evidence-based transfusion practice: an intervention development protocol. Implementation Science 2014, 9:92 http://www.implementationscience.com/content/9/1/92

14. Eldridge SM, Ukoumunne OC, Carlin JB (2009) The intra-cluster correlation coefficient in cluster randomized trials: A review of definitions. International Statistical Review, 77(3): 378-394

15. Ivers N, Jamtvedt G, Flottorp S, Young JM, Odgaard-Jensen J, French SD, O'Brien MA, Johansen M, Grimshaw J, Oxman AD: Audit and feedback: effects on professional practice and healthcare outcomes. Cochrane Database Syst Rev 2012, 6:CD000259.

16. Carver CS, Scheier MF. Control theory: a useful conceptual framework for personality-social, clinical and health psychology. Psychological Bulletin, 1998, 92, 111e135.

17. Michie S, Johnson A, Abraham C, Lawton R, Parker D, Walker A: Making psychological theory useful for implementing evidence based practice: a consensus approach. Qual Saf Health Care 2005, 14:26-33.

18. Moore et al Moore, G. F., Audrey, S., Barker, M., Bond, L., Bonell, C., Hardeman, W., & Baird, J. (2015). Process evaluation of complex interventions: Medical Research Council guidance. bmj, 350, h1258

19. Bellg AJ, Borrelli B, Resnick B, Hecht J, Minicucci DS, Ory M, Ogedegbe G, Orwig D, Ernst D, Czajkowski S: Enhancing treatment fidelity in health behavior change studies: best practices and recommendations from the NIH Behavior Change Consortium. Health Psychol 2004, 23(5):443.

20. Borrelli B. The assessment, monitoring, and enhancement of treatment fidelity in public health clinical trials. J Public Health Dent. 2011;71:S52–S63

21. Campbell MK, Elbourne DR, Altman DG. CONSORT statement: extension to cluster randomised trials. BMJ. 2004;328:702–708. doi: 10.1136/bmj.328.7441.702.

22. Huth EJ (1986). Guidelines on authorship of medical papers. Annals of Internal Medicine, 104, 269-274.

23. Glass RM (1992). New information for authors and readers. Group authorship, acknowledgements and rejected manuscripts. Journal of the American Medical Association, 268, 99.

24. Kempthorne, O. (1952). The design and analysis of experiments. Wiley; Chapman & Hall.

Appendix A: Collaborators and grant co-applicants

The AFFINITIE Programme is in association with:

AFFINITIE Grant Co-Investigators and collaborators	Ms Alison Deary, Head of Clinical Operations, NHS Blood and Transplant Clinical Trials Unit<br><br>Professor Jillian Francis, School of Health Sciences, City University London<br><br>Dr Liz Glidewell, Academic Unit of Primary Care, University of Leeds<br><br>Dr Natalie Gould, School of Health Sciences, City University London<br><br>Professor Jeremy Grimshaw, Ottawa Hospital Research Institute, University of Ottawa<br><br>Dr Fabiana Lorencatto, School of Health Sciences, City University London<br><br>Professor Susan Michie, Psychology, University College London<br><br>Dr Maria Prior, City University London<br><br>Dr Megan Rowley, NHS Blood and Transplant
Trial Steering Committee Chair	Dr Derek Norfolk, Retired Consultant Haematologist
Patient and Public Involvement Chair	Mr Alan White, member and past Chair of the Royal College of Physicians Patient and Carer Liaison Group (RCP-PCLG). Past Chair of an NHS Trust, and involvement as a patient representative (National Comparative Audit of Transfusion, the Intercollegiate Committee on Haematology

Appendix B: Sites involved in intervention development

The following sites were involved in the development of the intervention. These sites will be invited to participate in the national audit but will not be included in the randomised evaluation of the feedback as they have already been exposed to the intervention. These sites will therefore be given the enhanced feedback intervention:

1. Royal Bristol Infirmary, United Hospitals Bristol NHS Foundation Trust
2. Royal United Hospital Bath, Royal United Hospital Bath NHS Trust
3. Worthing Hospital, Western Sussex Hospitals NHS Trust
4. William Harvey Hospital, East Kent Hospitals NHS Foundation Trust

Appendix C: Regional Transfusion Committees

1. East of England RTC
2. East Midlands RTC
3. London RTC
4. North East RTC
5. North West RTC
6. South Central RTC
7. South East Coast RTC
8. South West RTC
9. West Midlands RTC
10. Yorkshire and the Humber RTC
11. Scotland
12. Wales
13. Northern Ireland
